# Alternative Algebraic Perspectives on CO/H_2_ PROX over MnO_2_ Composite Catalysts

**DOI:** 10.1021/acs.jcim.5c00072

**Published:** 2025-05-02

**Authors:** Marco Bertini, Francesco Ferrante, Laura Gueci, Antonio Prestianni, Dario Duca, Francesco Arena, Dmitry Yu. Murzin

**Affiliations:** † Dipartimento di Fisica e Chimica “Emilio Segrè”, Università degli Studi di Palermo, Viale delle Scienze Ed. 17, Palermo I-90128, Italy; ‡ Dipartimento di Ingegneria, Università degli Studi di Messina, Contrada di Dio, Messina I-98166, Italy; § Laboratory of Industrial Chemistry and Reaction Engineering, Johan Gadolin Process Chemistry Centre, Åbo Akademi University, Henriksgatan 2, Turku/Åbo 20500, Finland

## Abstract

This study presents a graph-based approach to investigate the steady-state kinetics of the preferential CO oxidation process in H_2_ (PROX) occurring on a MnO_2_ model fragment with manganese centers at varying oxidation states, simulating the surface Mn­(IV) active sites of a composite MnO_2_–CeO_2_ catalyst previously used in experimental applications. A novel modeling approach, termed DFT graph-based kinetic analysis (DFT-GKA), is introduced. It utilizes free activation energy (Δ*G* ^⧧^) values to characterize linear elementary events, supposed at pseudosteady-state, in this complex reaction system, as determined through density functional theory (DFT) integrated by thermochemical calculations. The implementation of this model is achieved using a *homemade* Common Lisp code, specifically designed for efficient manipulation of long lists essential for the analysis. Finally, the comprehensive ab initio DFT kinetic descriptors related to the CO/H_2_ PROX catalytic process on the manganese oxide fragments are discussed, highlighting their significance for future research and applications.

## Graphical abstract

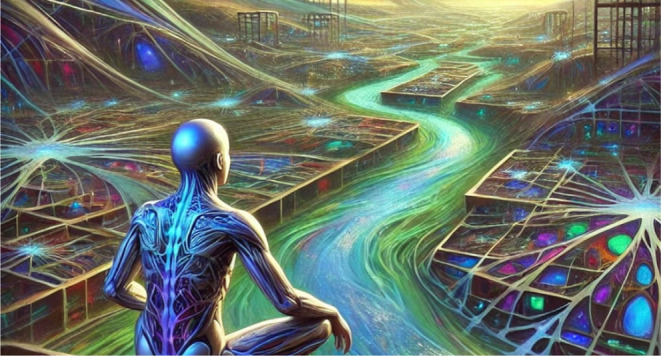

## Introduction

Carbon monoxide in hydrogen (CO/H_2_) preferential oxidation (PROX) is a process in which CO, in hydrogen rich feedstocks, is selectively oxidized to CO_2_ over H_2_ oxidation to H_2_O. It is pivotal in clean energy and emission control and serves as a key method for purifying hydrogen-rich streams for fuel cells and other technologies,^1^ particularly addressing toxicity and pollution challenges.^2^ A series of studies on CO/H_2_ PROX systems have been recently reported in the literature. These studies were focused on experimental investigations combined to a detailed density functional theory (DFT) analysis, conducted on model manganese fragments Mn_4_O_8_, Mn_4_O_7_, and Mn_4_O_9_, mimicking a highly dispersed manganese dioxide (MnO_2_) phase of a nanocomposite MnCeO_*x*_ system.^3,4^ In fact, experimental findings suggested a negligible role of the CeO_2_ promoter in the PROX reaction network of the composite MnCeO_*x*_ catalyst.^3–5^ However, “spillover” effects could occur at higher threshold temperatures, potentially enabling the transfer of atomic oxygen species from CeO_2_ to the manganese oxide fragments, thereby altering their oxidation state.^5^ Theoretical and experimental studies of these systems, at both macro- and microkinetic levels, have revealed a complex mechanism in which CO and H_2_ oxidation processes should influence each other. This necessitates model simplifications that reproduce conditions where such interactions are rendered almost negligible.

The oxidation processes of CO and H_2_ were already treated as independent, both at macro- and microkinetic levels, using ordinary differential equations (ODEs) and the simplified Christiansen method (SCM),^3–8^ respectively. In passing it is here recalled that the latter is based on the Christiansen method, as described in ref 9, where algebraic tools are used to determine catalytic cycle^10^ rates and surface species concentrations while accounting for their intrinsic fluctuations.^9^ These fluctuations, arising from adsorption–desorption and reaction processes, average out over time, leading to an effective steady-state condition that governs the macroscopic reaction rate.^3–9^

Despite the inherent approximations in treating the two oxidation processes as independent, the experimental results showed good correlation with both micro- and macro-kinetic models, which, as a consequence, were also in mutual agreement.^3–5^

The analysis proposed here employs a pseudosteady-state approach based on graph theory,^11,12^ utilizing a DFT-based kernel to obtain thermodynamic information, which is then used to derive kinetic descriptors via either the Eyring–Polanyi equation^6,13^ or, as will be explained later, a newly introduced related formulation. This analysis, in any case considering linear elementary steps, is grounded in the foundational perspective, where the overall reaction rate of a chemical transformation can be determined by the individual rates of its fundamental pathways, much like a vector is defined by its components relative to the coordinate axes.^10^

For chemists, it is intriguing to observe that the term graph, while rooted in Euler’s algebraic approach to solving the Königsberg bridge problem,^14^ was formally introduced by the mathematician Sylvester.^15^ Interestingly, Sylvester acknowledged that the term was inspired by chemical themes,^15^ specifically referring to the early Kekuléan *chemicograph* representations. This historical connection underscores the deep and enduring relationship between graph theory and chemistry. In fact, although graph theory has found applications across a wide range of disciplines—from engineering and psychology to economics and biology^10^ —its connection to chemistry has always been particularly profound.^16,17^ In recent years, this relationship has grown even stronger, with graph-theoretical approaches being employed to analyze complex networks^18^ of chemical interest.^19^

For instance, recent studies have focused on the analysis of chemical reaction networks (CRNs) in out-of-equilibrium systems using randomly generated graphs.^20^ These methods have proven invaluable in understanding the dynamic behavior of such systems, which are often characterized by nonlinear interactions and emergent properties. Moreover, graph theory has been instrumental in the study of electrochemical reaction networks, particularly in the context of energy storage and disposal.^21^ These networks play a critical role in advancing battery technologies, as they help characterize interfacial reactions^22^ that are fundamental to the development of efficient and sustainable electrochemical systems.

Notably, the link between graph theory and the study of enzymatic and heterogeneous catalysis dates back almost five decades, representing the first attempt to study reaction network by using graph theory. The chemical graph approach to catalysis originally introduced to study enzymatic reactions was actually established in the original work of King and Altman.^23^ In order to improve the algorithmic efficiency, similar graph-based approaches were later applied to the same enzymatic field in modified versions,^24^ utilizing analogies derived from the graph-based Mason model^25^ employed for the analysis of complex electric circuits. Temkin proposed also a straightforward graph-based method to determine the number of independent routes, formed by linear steps, present in a complex heterogeneous catalytic mechanism,^26^ drawing on the theoretical insights of Horiuti.^27^

In recent years, the chemical graph approach to catalysis has been proposed as a powerful tool for analyzing thermodynamic^28^ and kinetic^27^ descriptors of heterogeneous catalytic systems, including those exhibiting nonlinearity in their mechanism steps,^29^ even through purely theoretical methods.^30^ Applications related to the latter, along with methods for determining the distinct pathways of a complex mechanism will form the core methodology employed in this work.

The present study will, in particular, reference theoretical aspects of the aforementioned PROX systems,^3–5^ demonstrating how their reconfiguration and application in a graph algebraic context,^10,27^ consistent with the classical approaches, by e.g., Christiansen,^9^ Horiuti and Nakamura^31^ and Temkin,^26^ can offer new perspectives and interpretations in the rationalization of the CO PROX reaction on composite MnO_2_–CeO_2_ catalysts, also documenting large applicability of graph theory to heterogeneous catalytic reactions.^10,27,30^

The following section outlines the computational methodologies employed to derive the fundamental thermodynamic data necessary for the graph kinetic analysis of the catalytic PROX reaction. The kinetic analysis, along with the discussion of key results, will be presented in a dedicated section. Between these two sections, a third one, subdivided into multiple subsections, will introduce the development of the DFT-GKA—graph kinetic analysis via DFT—model and the *GCODE* tool that enables its implementation. The model presentation begins with basic principles. In this context, approaches are initially proposed to simplify the identification of catalytic cycles within polycyclic mechanisms, which consist primarily of elementary steps involving surface intermediates. The description of the machinery useful to obtain kinetic descriptors such as activity and selectivity descriptors of heterogeneous catalytic reactions and particularly of the title PROX reaction will thus follow. The *homemade GCODE*, available on *GitHub* at https://github.com/enonmorferehwon/gcode, is finally presented and proposed as a means to make DFT-GKA a practical modeling tool, complementing laboratory research on heterogeneous catalysis. To illustrate its functionality, the title reaction serves as a case study, with key kinetic descriptors calculated accordingly. We hope this example encourages wider adoption of the approach, dispelling its reputation as an exceedingly abstruse method.

## Computational Details

The reported density functional theory, DFT, data regarding structural and energetic properties of all the intermediate, i.e., minima, and transition state, TS, species involved in the surface processes, as reported in ref 4, were derived by calculations using the M06-L exchange-correlation functional,^32^ combined with the correlation consistent polarized valence double-ζ, cc-pvdz, basis set for light atoms,^33^ and the relativistic small core Stuttgart ’97 basis set with effective core potentials for manganese.^34^ The graph theory approach was implemented according to the methodologies described by Yablonskii et al.^10,27^ Details on the approach will be given in the next section where will be also explained the origin of the occurrence probabilities for the elementary events of the different surface intermediates. The occurrence probabilities for the transformation of the latter are determined using the free energy differences characterizing them and the corresponding TSs, that is, using the free activation energy, Δ*G* ^⧧^, values. The free energies of both the surface species and transition states were calculated starting from DFT electronic SCF energies; vibrational partition functions were obtained from the DFT harmonic frequencies, while translational and rotational contributions were evaluated by means of semiclassical formulas. All thermodynamic quantities, hence Gibbs free energies, at all the desired temperatures were then derived by applying standard statistical thermodynamics laws—see for example Ochterski^35^ for a detailed description of their implementation in electronic structure codes—excepting for entropy, to which the quasi-harmonic correction proposed by Grimme,^36^ was applied, according to which the contribution of low-lying vibrational modes to the entropy (a frequency cutoff of 100 cm^–1^ was used in this work) is replaced by a corresponding effective rotational entropy. The Goodvibes code^37^ was used for deriving thermodynamic quantities with the correction above. With respect to this, it is interesting to recall that the accuracy of M06-L^32^ on determining barrier heights was tested on several reactions involving transition metals and it could be quantified on the base of an averaged mean unsigned error of ca. 10 kJ mol^–1^.^38,39^

## Graph Model

### Bases

The schematic reaction pathway for the CO/H_2_ PROX process on MnO_2_–CeO_2_ catalysts coming from theoretical studies and in agreement with the experimental findings^5^ is shown in Figure 1. The theoretical approaches, in particular, involved the modeling of a Mn_4_O_8_ fragment, which rearranged during the catalytic transformation into the hypo- and hyper-oxygenated species, Mn_4_O_7_ and Mn_4_O_9_.^3–5^ In Figure 1, these species correspond to σO, σ and σO_2_, respectively. In the same figure, I2 and I3 are conversely pseudoisomer species^40^ whose elemental composition can be derived from that of σO, adding to it the molar mass of CO. Similarly, I6 and I7 are pseudoisomers obtained by adding a CO molecule to the fragment σO_2_ while the group of pseudoisomers I8–I12 by adding H_2_ to σO_2_.

**Figure 1:**
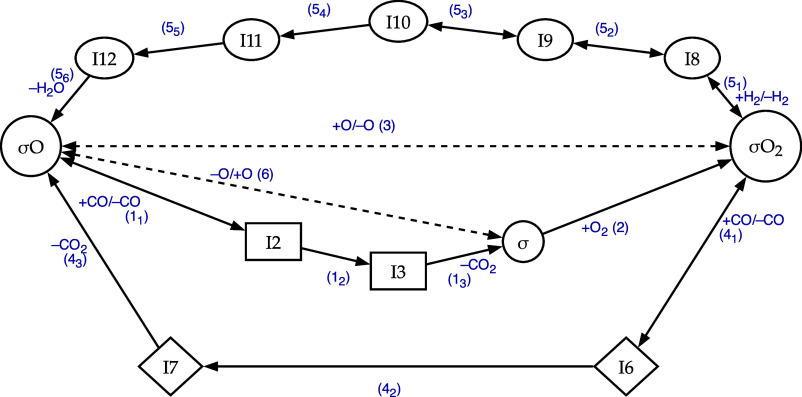
CO PROX reaction path on MnO_2_ fragments determined in the frame of a DFT approach.^3–5^ The *N*th surface intermediates are represented as IN, being *N* the notation already used in the references above. Species σO (Mn_4_O_8_), σ (Mn_4_O_7_) and σO_2_ (Mn_4_O_9_), would be synonym of I1, I4 and I5 hence correspond to species 1, 4, and 5 in refs 3–5 . The remaining species are pseudoisomers,^40^ grouped into nodes with distinct box shapes. Each shape corresponds to a specific pseudoisomer group. Single and double arrows represent irreversible and reversible steps. Dashed steps (3) and (6), which will not be deepened with respect of their energetics and kinetics in this study, correspond to spillover phenomena that are activated above a threshold temperature. The residual steps and their associated species are fully characterized thermodynamically and kinetically. Adsorbing and desorbing species are easily identified by analyzing the behavior of the corresponding catalytic cycles.

In Figure 1 the set of steps (*N*_*n*_), e.g., (1_1_), (1_2_), (1_3_), individuate collective-steps (*N*), e.g., (1), in which are involved single families of pseudoisomers. Collective steps (i.e., contracted edges), singly including pseudoisomers and the molecular steps in which they are involved, are employed in Scheme 1. This represents a homeomorphic graph^17^ corresponding to the one illustrated in Figure 1. Dashed lines, in Scheme 1 and in Figure 1, denote spillover phenomena whose energetics and kinetics are beyond the scope of this study.

**Scheme 1:**
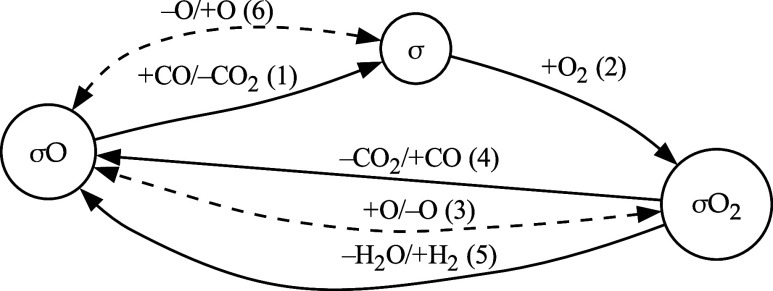
Simplified Representation of the Whole PROX on MnO_2_–CeO_2_ Catalysts: Mn_4_O_8_ ≡ σO, Mn_4_O_7_ ≡ σ, Mn_4_O_9_ ≡ σO_2_ ^a^

a Notation used is consistent with that of graph theory applied to catalytic reactions; size of the nodes and the length of the edges have no physical significance. The numbers in parentheses identify collective-steps, involving pseudo-isomers belonging to the same FIEMs^41^ that characterize the catalytic cycles in the whole mechanism.

An in-depth analytical study of the characteristics of steps (3) and (6) is, indeed, currently underway. The study aims to unravel the role of these steps in the overall reaction mechanism that, as will be mentioned later, are representative of Mars–van Krevelen (MvK) processes,^42^ assisted by lattice oxygen atoms of the CeO_2_ phase. Groups of pseudoisomers are termed family of isomeric ensembles of molecules (FIEM).^41^

In Figure 1, to distinguish different FIEMs, their components are enclosed within square, diamond, or oval frames. Within any given family, the pseudoisomers interconvert without involving species from other phases such as, in the present case, the gas-phase. For each step not involving spillover (solid line in Figure 1), Table S1, in the Supporting Information, provides details concerning the probability of occurrence of any event, π_ev_, taking place on a catalytic active surface unit^43^ and corresponding to a stated step *N*_*n*_

1 $$\pi_{ev}=\exp\left(-\frac{\Delta G^{⧧}}{RT}\right)$$

*R* and *T* are the ideal-gas constant and a given temperature value. Δ*G* ^⧧^ is the free activation energy of a given surface event, ev. It denotes the change in Gibbs free energy, determined here by using DFT methods and the standard formulas of statistical thermodynamics, as described in the Computational Details. The Δ*G* ^⧧^ values straightforwardly represent the energy barriers separating any surface intermediate from the adjacent transition state (TS) along the reaction path coordinate. The relative position of TSs—preceding or following the intermediate—depends on whether the step is forward or backward. We recall here that, for the investigated system, the adsorption (desorption) processes are not characterized by a barrier corresponding to a transition state, so activation free energy is equal to the adsorption (desorption) free energy when it has a positive value, or to zero when it is negative.^4^

In the following, π_ev_ will be set equal to 0 when its value is less than 10^‑20^—at 298 K, this corresponds to a Δ*G* ^⧧^ value of ca. 114 kJ mol^–1^. In fact, at the largest temperature here considered, the corresponding event step rate would result ca. 10^‑7^s^–1^, which is a very low value for a surface intermediate transformation on a catalytic site. In this case the corresponding step does not occur, determining irreversibility conditions. The Eyring–Polanyi equation written in the form^44^

$$k_{ev}=\kappa \frac{k_{B}T}{h}\exp\left(-\frac{\Delta G^{⧧}}{RT}\right)=\kappa \frac{k_{B}T}{h}\pi_{ev}$$ 2

allows one to evaluate the rate constant *k*_ev_ [s^–1^]. For molecular surface reactions at given temperatures, this is the frequency of occurrence of an event normalized to the surface molar ratio of the sites involved—or, alternatively, the elementary surface reaction rate for a unit concentration of the reacting intermediate.^10^ For this reason, in the following, it will be also called event (or step) rate, or simply rate. In this, *k*_B_ and *h* are, in the order, the Boltzmann and Planck constants. The transmission coefficient κ, which represents the probability that a system crossing the transition state will proceed to form products rather than reverting to reactants, is approximated to 1. This is consistent with the original formulation of the transition state theory^13^ and aligns with recent applications to heterogeneous catalysis.^3–8,30,45^

At this point, it is important to emphasize that eq 2 allows for the evaluation of catalytic transformations exclusively regarding surface species. In other words, the equation above for the title system enables the assessment of transformations of species that have already been adsorbed from the gas-phase onto the catalyst surface, by just linear steps.

However, to evaluate the rates of the steps involving adsorption, as an example the step (1_1_) of Figure 1, besides the adsorption probabilities, which could be identified with the corresponding π_ev_ values it is also necessary to take into account the hitting frequency of the gas-phase species on the catalytically active surface unit.^40,46,47^ This need is driven by the presence of multiple gas-phase species, g, competing for the same catalytic sites. In fact, at a given temperature, the hitting frequency (*f* [s^–1^]) is related to the partial pressure (*p*) and the molecular mass (*m*) of the different gas-phase species: $f_{g}\propto p_{g}/\sqrt{m_{g}}$ .^40,46–49^ The latter hence affects the adsorption of the different molecules undergoing surface transformations and reactions. In this study, steps (1_1_) and (4_1_), as well as (2) and (5_1_), involve adsorption: CO in the former two and O_2_ and H_2_ in the latter two.

The model summarized in the graph of Figure 1 considers a catalytically active site unit undergoing transformation under pseudosteady-state conditions, with an approximately constant gas-phase composition. This can be deduced from the partial pressures of the different gas-phase species, g, and is appropriately expressed in terms of their mole fractions, χ_g_. The hitting probability of molecules, γ, at a given temperature can be conversely expressed as its hitting frequency, *f*_γ_, normalized to the hitting frequencies of all gas-phase species, *f*_g_, involved in the process. This ratio, *F*_γ_ = *f*_γ_/∑_g_ *f*_g_, represents the number of collisions by molecules γ relative to the total number of molecular collisions on the surface. Following these inferences, it is possible to reformulate the rate of steps involving adsorption, using an equation that takes into account the partial pressure of the adsorbing reagents

$$k_{ev}^{\gamma }=\kappa \frac{k_{B}T}{h}F_{\gamma }\exp\left(-\frac{\Delta G^{⧧}}{RT}\right)=\kappa \frac{k_{B}T}{h}\pi_{ev}^{\gamma }$$ 3

This equation finds justification because eq 2 generally express a probability per unit of time.^40^ In the case of adsorption, it represents the probability that a gas-phase species will be absorbed upon contact with a given active surface unit. *F*_γ_, in turn, represents the hitting probability of a gaseous species in a gas mixture.

Their product, therefore, is the probability per unit of time that the species will initially collide and then be adsorbed on a surface.^47^ The use of eq 3, for steps involving adsorption, offers an additional advantage related to its dimensional consistency that actually aligns with the one of eq 2. In the former the probability π_ev_^γ^ has been introduced to simplify the representation of the rate descriptor *k*_ev_^γ^ and to highlight the similarity between eqs 2 and 3.[Fn fn1] Furthermore, since it is possible to write $F_{\gamma }=\exp\left(-\frac{\varepsilon_{\gamma }}{RT}\right)$ , where ϵ_γ_ represents a generic energetic factor, we can set

$$\pi_{ev}^{\gamma }=\exp\left(-\frac{\Delta G^{⧧}+\varepsilon_{\gamma }}{RT}\right)$$

and thus correct the energy barriers of the adsorption processes by defining Δ*G*_γ_^⧧^

4 $$\Delta G_{\gamma }^{⧧}=\Delta G^{⧧}+\varepsilon_{\gamma }=\Delta G^{⧧}-RT\cdot \ln\left(F_{\gamma }\right)$$

This allows us for an energy correction, ϵ_γ_, in the form expressed in eq 4 due to the partial pressure and molecular mass of the adsorbing gas-phase species, γ, to be directly applied to the Δ*G* ^⧧^. At variance, the latter straightforwardly represents the adsorption probability when only one gas species is present. Since the logarithm value is always less than or equal to one, the correction delays the involved step or, at most, has no effect.

To evaluate the significant effects of the partial pressure exerted by the components of a gas mixture, Table 1 presents the ϵ_γ_ corrections to the Δ*G* ^⧧^ values, in order to state the Δ*G*_γ_^⧧^ terms corresponding to the adsorption of different components from various gas mixtures of interest, at two temperatures: 373 and 523 K.

**Table 1:** DFT Energy Barriers (Δ*G* ^⧧^) Involved in the Adsorption Steps (S) of Single Molecule (CO, O_2_, H_2_) on Different MnO_2_ Fragments (σO, σ, σO_2_) and Relative Corrections (ϵ_γ_) at Different Temperature (*T*) and Partial Pressure of CO, O_2_, H_2_, N_2_

			gas mixture composition^a^			
			(CO O_2_ H_2_ N_2_)			
S^b^	adsorption events on MnO_2_ fragments	DFT^c^	(1 1 98 0)	(1 1 49 49)	(5 1 49 45)	(1 5 49 45)
		Δ*G* ^⧧^/kJ mol^–1^	ϵ_γ_/kJ mol^–1^			
(1_1_)	CO + σO → I2	27.0|50.1	18.3|25.7	16.9|23.7	11.9|16.7	16.9|23.7
(2)	O_2_ + σ → σO_2_	0.0|0.0	18.5|26.0	17.1|24.0	17.1|24.0	12.1|17.0
(4_1_)	CO + σO_2_ → I6	17.3|41.9	18.3|25.7	16.9|23.7	11.9|16.7	16.9|23.7
(5_1_)	H_2_ + σO_2_ → I8	11.5|25.4	0.0|0.0	0.8|1.1	0.8|1.1	0.8|1.1

a A total pressure of 100 kPa was considered; the gas mixtures contained CO, O_2_, H_2_, and N_2_ having percentage individuated by the tuples of the title columns: (1 1 98 0), (1 1 49 49), (5 1 49 45), and (1 5 49 45). The vertical bar distinguishes the corrections at the two considered temperature: 373 on the left and 523 K on the right.

b For the meaning of the acronym refer to caption of Figure 1.

c The Δ*G* ^⧧^ values were determined using DFT approaches, employing the Grimme quasi-harmonic correction method^36^ and the Goodvibes code.^37^ The pressure of the species involved in the adsorption was set to 100 kPa.

Pseudosteady-state conditions were assumed for the system while just linear steps, namely the adsorption of individual species and the transformation of surface pseudoisomers, were always involved in the model.

Steps (3) and (6) involving lattice oxygen species potentially supplied by CeO_2_ to Mn centers, as is explained in the Supporting Information discussing Scheme S1, are also assumed to be at equilibrium, acting the CeO_2_ phase as a sort of atomic oxygen buffer, activated above a threshold temperature. The assumptions introduced and the considerations done should ensure linear conditions within the studied system. While the competitive adsorption of H_2_ and CO on the σO_2_ site could present a challenge in this regard, the significant disparity in the rates of these two processes—observed both experimentally and computationally^3–5^ —should serve as a stabilizing factor, maintaining at least quasi-linear conditions. The representation of the information into Figure 1 is thus framed in the graph-theory paradigm initially proposed by King and Altman^23^ and revisited up to recently in excellent monographes on the applications of the kinetics machinery to catalysis by Yablonskii et al.^10,27^ This approach allows for a schematic simplification of the overall pseudosteady-state mechanism representation without losing any information regarding the thermodynamics and especially the kinetics of the system. A further simplification still arises grouping consecutive steps involving just pseudoisomers or rather steps starting from and arriving to corresponding pseudoisomers. In this way, condensed collective-steps are clearly formed. Scheme 1 actually shows this simplified representation of the whole mechanism illustrated by Figure 1.

It is interesting to note that only the surface species related to the manganese fragments of the model catalyst, i.e., σO, σ, σO_2_, in their different oxidation states, Mn_4_O_8_, Mn_4_O_7_, Mn_4_O_9_, persist in this reduced representation. Plus and minus signs associated with the gaseous species (CO, CO_2_, H_2_, H_2_O) as well as with the atomic oxygen species, which potentially originate from CeO_2_, refer to the interactions of these species with the corresponding surface intermediates.

If even a single step involved in a given collective step is irreversible (see Table S1), the latter will also be irreversible. For this reason, steps (1), (2), (4), and (5) in Scheme 1 are all irreversible. In contrast, steps (3) and (6) when activated could be reversible due to their nature, which implicates the exchange of atomic oxygen between the CeO_2_ and MnO_2_ matrices.^5^ The irreversibility of the steps (1), (4) and (5) is reasonable because of strong changes in thermodynamic parameters (i.e., Δ*H* and Δ*S*) during conversion of CO and H_2_ into surface intermediates leading to CO_2_ and H_2_O products on the MnO_2_–CeO_2_ catalyst.^3,5^ Likewise, the step (2) is retained irreversible because of strong electronic interactions of O-vacant sites with gas-phase O_2_ and the high reactivity of the generated hyper-oxygenated σO_2_ sites toward CO, overall resulting in negligible changes of the catalyst surface under steady-state conditions.^5^

In order to reconstruct the whole reaction path it is necessary to identify the different cycles present in the same path and then combine the isolated cycles in such a way as to derive the information on the descriptors of interest. In the present case, information on catalytic activity and selectivity in the title PROX process, along with other catalytic descriptors will be obtained starting from the reduced graph of Scheme 1.

Recalling that, mathematically, a cycle is a closed loop in which vertices do not repeat except for the start and end,^50^ and using the already defined collective steps—say (*l*), (*m*), and (*n*)—of one cycle, we can formally represent it as {*l*,*m*,*n*}. From this and referring to Scheme 1, it can be inferred^3–5^ that the cycles, such as the {2,3,6}, which include step (3) and/or (6), are facilitated by parallel cyclic red-ox transformations, as depicted in Scheme S1, occurring, independently of those from Scheme 1, on CeO_2_ and inducing above a threshold temperature an exchange of atomic oxygen between CeO_2_ and MnO_2_ matrices.^5^ Below that temperature the cycles containing steps that involve atomic oxygen transfer, namely steps (3) and (6), would conversely become irrelevant to the overall mechanism.^3–5^ As explained in the Supporting Information, cycles involving steps (3) and/or (6) will be labeled as SMK, that is secondary MvK, cycles; the remaining ones as PMK, primary MvK, cycles. Given this, PMK and SMK processes should occur at lower and higher temperature, respectively.

A systematic kinetic analysis of a given process, particularly the one under examination, should begin by identifying the cycles that can be regarded as fundamental. These should be thought of as independent sets of steps whose combination defines other derived cycles within the reaction mechanism.^10,26^ Fundamental and derived cycles, thus identified, define a reaction space, in which their combinations individuate, as will be shown, the overall reaction mechanism of interest. It is important here to emphasize that “fundamental”, referred to cycles, is a term from mathematical language and should not be interpreted as “the most chemically significant”. Further, it is worth recalling that the cycles mentioned above are inherently simple and, as such, do not include repeated nodes.^10^

Referring to Scheme 1, cycles {1,2,3}, {1,2,4}, {1,2,5} and {1,6} can be identified as mathematically fundamental and orthogonal, where it is always possible to observe at least one edge not contained in any other set. In chemical terms, these cycles may be regarded as independent pathways or basic routes.^10^

It is not necessary to search for others, considering that the number of fundamental cycles must satisfy the equation *C* = *S* – *I* + 1,^10,41^ where *C* is the number of fundamental cycles, and *S* and *I* the number of steps and surface intermediates, respectively present in the overall mechanism. Notably, grouping the pseudoisomers instead of listing each one individually in every cycle does not impact the calculation of the number of cycles or their intrinsic relationships. In fact, removing an edge between two pseudoisomer nodes in a cycle, one by one, and replacing the original two nodes with a single grouped node, thereby closing the cycle again, reduces the cycle by one node and one edge at a time. This clearly leaves the total number of fundamental cycles unchanged.

Applying the rules of the symmetric difference,^51^ which for two generic sets S1 and S2 is formally defined as S1△S2 = (S1∪S2)\(S1∩S2), to all pairs of fundamental sets of steps—and subsequently, iteratively, to all pairs of derived and fundamental sets—yields all derived cycles. The derived cycles, along with the fundamental ones, can be thus used for the study of the overall reaction mechanism.

For the present system, 15 cycles were identified. Besides the fundamental ones indexed as C_1_ ≡ {1,2,3}, C_2_ ≡ {1,2,4}, C_3_ ≡ {1,2,5}, C_4_ ≡ {1,6}, the remaining physically significant were: C_5_ ≡ {2,5,6}, C_6_ ≡ {2,4,6}, C_7_ ≡ {2,3,6}, C_8_ ≡ {3,5}, C_9_ ≡ {3,4}. Details on these cycles and on the corresponding elementary steps forming them are given in Table 2.

**Table 2:** Surface Events, Horiuti Numbers and Other Information Concerning the Cycles in the Title PROX

S^a^	surface event on MnO_2_ fragments^b^	C_1_ ^c^	C_2_	C_3_	C_4_	C_5_	C_6_	C_7_	C_8_	C_9_
(1)	CO + σO → CO_2_ + σ	1	1	1	1	0	0	0	0	0
(2)	O_2_ + σ → σO_2_	1	1	1	0	1	1	1	0	0
(3)	O + σO ⇌ σO_2_	–1	0	0	0	0	0	–1	1	1
(4)	CO + σO_2_ → CO_2_ + σO	0	1	0	0	0	1	0	0	1
(5)	H_2_ + σO_2_ → H_2_O + σO	0	0	1	0	1	0	0	1	0
(6)	O + σ ⇌ σO	0	0	0	1	–1	–1	–1	0	0
		nc	c	c	c	nc	nc	nc	c	c
		SMK	PMK	PMK	SMK	SMK	SMK	SMK	SMK	SMK

a S is any condensed collective-step in the reaction Scheme 1.

b Steps (3) and (6) are consistent with mechanisms involving CeO_2_. These steps are indeed ruled by the cycle shown into Scheme S1.

c Cycles C_1_–C_9_ characterize the reactivity of manganese oxide when simulated by Mn_4_O_8_ fragments. The different cycles are obtained by multiplying the Horiuti numbers^10,27^ of a given column by the corresponding steps and summing the results together. Primary and secondary Mars–van Krevelen, PMK and SMK processes involve and do not involve O species originating from CeO_2_ fragments, respectively. Terms c and nc summarize canonical and noncanonical cycle reaction paths. The former, unlike the latter, do not involve O atomic species among the products.

Other sets, {4,5}, {2,3,4,5,6}, {1,4,5,6}, {1,2,3,4,5}, {1,3,4,6}, {1,3,5,6}, are not indexed and not further considered because either physically inconsistent as cycles, given the here assumption on the irreversibility of some basic steps, i.e., set {4,5}, or obtained as linear combination of the already indexed cycles, i.e., all the remaining sets. It is evident that C_7_ does not involve the formation either of H_2_O or CO_2_. Cycles C_5_ and C_8_ result instead in the formation of H_2_O. In all the others, CO_2_ is produced. Specifically, in cycle C_2_, two molecules of CO_2_ are produced. Apart from the two previously mentioned cycles, H_2_O is also produced in C_3_. Cycles C_2_, C_3_, C_4_, C_8_, C_9_ can be considered as canonical, c, stoichiometric process because does not involve surface species as products. The remaining ones complementarily are not canonical, nc, cycles because involve the formation of oxygen surface species that will need to be eliminated or transformed in a subsequent stage. It should be noted that the cycles labeled as nc[Fn fn2] are usually not included in the Horiuti–Temkin theory framework,^10,53^ which implicitly underpins the kinetic model proposed here. Within this framework, the linear dependence inherent in the elementary surface reactions is systematically extended to the overall catalytic reactions,^53^ furthermore ensuring the absence of surface intermediates in the final products.

The combination of the Horiuti numbers^10,27^ with the surface events, both present in Table 2, allows for the reconstruction of the different cycles taking place on the Mn_4_O_8_ manganese fragments. As an example, in the following, the C_2_ and C_3_, c PMK, cycle stoichiometric reactions as obtained by using the Horiuti numbers^10,27^ of Table 2 are given

process 1 $$2CO+O_{2}\to 2{CO}_{2}$$

process 2 $$CO+H_{2}+O_{2}\to {CO}_{2}+H_{2}O$$

The information on the reciprocal interaction of the cycles, necessary to compute e.g., either coupling or rate-retardant parameters useful to reconstruct a complex polycyclic reaction mechanism,^27^ force to go back to the representation of Figure 1. It has to be recalled that the steady-state rate, *r*_[s]_, of one or more sequential steps, s, in a polycyclic mechanism is, as stated in eq 5,^10,27^ the sum of the rates (i.e., the sum of the product of the rates of the elementary event steps) characterizing each cycle *C*_[s]*i*_ belonging to the set of *n* cycles that originate from the polycyclic system and contain the steps s, multiplied by its corresponding coupling parameter, α_*i*_, and divided by the sum of all the rate-retardant components, *D*_*j*_ ^27^

5 $$r_{\left[s\right]}=\frac{\sum_{i}^{n}C_{\left[s\right]i}\alpha_{i}}{\sum_{j}D_{j}}$$

Individual terms, *D*_*j*_ or α_*i*_, are associated with the “node” spanning tree (Σ-T) and the coupling tree (X-T) graphs while *C*_[s]*i*_ with homonym cyclic graphs.[Fn fn3] Both Σ-T and X-T graphs, like the cycles *C*_[s]_, are subgraphs within the overall graph under analysis. Specifically, X-Ts represent the branching factor of given cycles. It has to be emphasized that α_*i*_ is not related to a single subgraph connected to the cycle *C*_[s]*i*_; rather, it is associated with a set of X-T subgraphs, each individually coupled to that cycle^10^ (see eq 9 for further details). The characteristics of Σ-T, X-T and *C*_[s]_ subgraphs will be detailed in the following dedicated sections. Both Σ-T and X-T graphs by definition do not contain any cycles. Respecting the reversibility rules of the mechanism considered Σ-Ts connect all the nodes within a graph while X-Ts join all steps of a polycyclic system that do not belong to a given cycle (within the considered polycyclic system) to that cycle.^27^ Clearly, the latter are absent in single cycle mechanism containing given steps s

6 $$r_{\left[s\right]}=\frac{C_{\left[s\right]}}{D}$$

with *D* = ∑_*j*_ *D*_*j*_. In this case, the corresponding kinetics could be treated applying the SCM approach.^3–8^ The Σ-T and X-T graphs are indeed used to asses two distinct aspects: the Σ-T graphs evaluate the effects of all possible step-chains involved in the entire mechanism, while the X-T graphs focus on the impact of each step not part of a specific cycle, on that cycle. These effects are actually summarized by terms *D*_*j*_ and α_*i*_, as outlined in eq 5.^10,27^

The lack of reversibility, as evidenced by Scheme 1, in the cycles not including steps (3) and (6) simplifies the representation and use of the cycles for the modeling applications here discussed. Indeed, focusing on the PMK low-temperature regime, it can be readily derived a simplified representation of the reaction network, as illustrated in Figure 2. This representation is simply achieved by eliminating steps (3) and (6), which pertain to the exclusion of the SMK cycles, previously detailed in Figure 1. Similar to the graph in Figure 1, the fully equivalent graphs (a) and (b) in Figure 2 are directed graphs, also known as oriented graphs, owing to the specific directionalities assigned to their edges. Further referring to Table 2, it is easy to recognize that the condensed cycles C_2_ and C_3_ represented by the simple stoichiometrically [Disp-formula fdstep_1] and [Disp-formula fdstep_2] are the only ones involved in the PMK mechanism.

**Figure 2:**
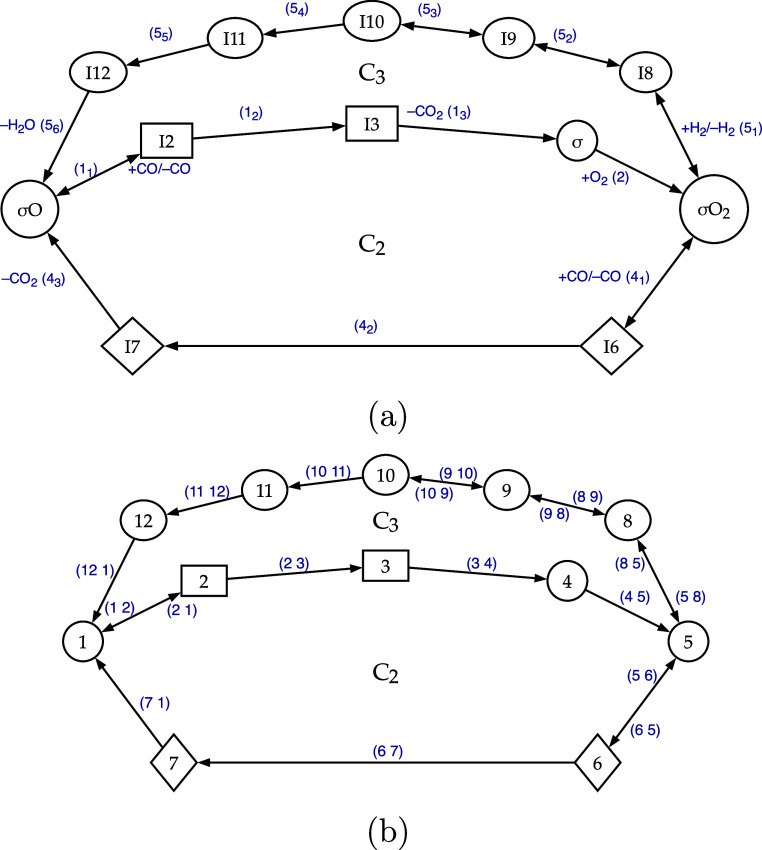
PMK reduced reaction path determined by DFT for the CO/H_2_ PROX occurring on MnO_2_–CeO_2_ mixed catalysts.^3–5^ C_2_ and C_3_ identify the corresponding cycles in Table 2. Graphs (a) and (b) are equivalent. The latter presents a more formal mathematical notation, employed in the subsequent sections, which emphasizes the connection between the nodes/reactants and their corresponding edges/reactions.

Referring to eq 5, note that in the system under consideration *n* = 1, while *C*_[s]_ correspond either to C_2_ or C_3_. In fact, due to their peculiar irreversibility, it is not possible to hypothesize any linear combinations between cycles C_2_ and C_3_. The detail shown in Figure 2b, omitting the explicit representation of the gas-phase, clarifies the notation used in the following discussion, which adopts a more mathematical formalism and enables direct comparison with previous works.^3–5^

### Σ-T Graphs: Springs, Wells and Ponds

In order to understand the origin of the spanning trees, Σ-T graphs, and their role in the kinetic analysis it is useful to discuss them, in a more formal way. Different species, corresponding to nodes of the graph, will be identified by numbers, while elementary events, corresponding to weighted edges of the graph, could be represented as tuples of three numbers. In the tuples, the first two numbers identify, in the order, surface reactants and products, while the third value is related to an activation energy parameter—such as the activation free energy barrier, Δ*G* ^⧧^, between the reactant and the TS preceding the formation of the product—or, for an example, the related probability of occurrence (per unit of time) of the event involving the same energy parameter. An illustration of these tuples can be found in the Supporting Information. For simplicity, since either the energy or the corresponding event rate information can be introduced as needed, the mechanism shown in Figure 2 may alternatively be represented as a vector of two-dimensional simplified tuples.

These have been actually used by the *homemade* Common Lisp *GCODE*, described later in a dedicated section. The simplified tuples include only pairs of transforming species (nodes) and events involving them (edges), as illustrated by graph (b) in Figure 2

$$\begin{array}{l} (\left(1\;2\right)\left(2\;1\right)\left(2\;3\right)\left(3\;4\right)\left(4\;5\right)\left(5\;6\right)\left(5\;8\right)\left(6\;5\right)\left(6\;7\right)\left(7\;1\right)\\ \left(8\;5\right)\left(8\;9\right)\left(9\;8\right)\left(9\;10\right)\left(10\;9\right)\left(10\;11\right)\left(11\;12\right)\left(12\;1\right)) \end{array}$$

It is important to emphasize that this representation, like the one characterized by the three-dimensional tuples, allows for easy identification of the symmetric edges corresponding to reversible steps, namely the forward and backward steps, as an example the couple ((5 6) (6 5)). In passing, it should be noted that the species are labeled by the same identifiers already employed in ref 5.

It has already been said that Σ-T graphs must not contain cycles, but they must connect all the nodes, or species, without including any symmetric edge, or event. In Σ-Ts, multiple starting points, or springs, can be identified—i.e., nodes with out-degree ≠ 0 and in-degree = 0—but only one end point, or pond,^54^ has to be present. This is a node with out-degree = 0 and in-degree ≠ 0. Additionally, there cannot be nodes with out-degree ≥ 2 and in-degree = 0, such as wells with two exits and no entrance.

Ponds have a special role, as will be shown through the use of a metaphor. If the graph represents channels through which water flows, the pond collects this water, which arrives from the various springs either directly or through the filling and emptying of wells. Moving away from the metaphor and returning to a kinetic pathway, one can think of the corresponding Σ-T as one among the possible useful route for the formation of the species corresponding to its pond.[Fn fn4] This metaphor is confirmed by the graph algebra, particularly in the Mason signal-flow graph approach.^10,25,27^ In order to obtain graphs with the Σ-T characteristics, it is necessary to remove from the original polycyclic graph a number of edges, whether symmetric or not, equal to the number of independent cycles, in the present case 2. This allows to calculate, considering all pairwise combinations of edges to be removed, the maximum number of graphs with both symmetric and not symmetric edges; in this case this number is equal to $\left(\begin{array}{c} 13\\ 2 \end{array}\right)$ , which is 78 graphs. The number of spanning trees can be directly computed using any cofactor of the Laplacian (or Kirchhoff) matrix of the given undirected graph, as stated by Kirchhoff’s theorem. Recall that the Laplacian matrix is defined as the difference between the degree matrix and the adjacency matrix of the graph.^55^

Of the found graphs, those containing cycles can be excluded. In this regard, it should be noted that cycles in a graph remain when the excluded pairs of edges are located on lines of nodes between the ones that connect the same cycles, which in this case are nodes 1 and 5, corresponding to the species σO and σO_2_. Given that the paths connecting these nodes in the graph consist of 3, 4, and 6 steps (see Figure 2), and that 2 steps need to be eliminated—to generate each spanning tree, in this case—it is possible to calculate the number of spanning trees (containing one residual cycle) to be removed. These are calculated as $\left(\begin{array}{c} 3\\ 2 \end{array}\right)+\left(\begin{array}{c} 4\\ 2 \end{array}\right)+\left(\begin{array}{c} 6\\ 2 \end{array}\right)$ , reducing the number of significant graphs—which include both symmetric and asymmetric edges—to 54. However, since the Σ-T graphs of interest should not contain symmetric edges, each of the 54 acyclic graphs, but having symmetric edges, can give rise to more than one Σ-T. Specifically, each pair of symmetric edges will generate two related graphs, one characterized by the forward and the other by the corresponding backward step. This means that an acyclic graph with symmetric edges, lacking two edges compared to the graph representing the mechanism in Figure 2, will generate 2^*n*^ Σ-T graphs, if the original one contained *n* pairs of symmetric edges.

Figure 2 shows that the spanning trees of the represented graph can possess, at most, 5 reversible edges. However, not all of the 54 residual Σ-Ts have 5 reversible edges (potentially having 5, 4, or 3 instead), making it challenging to predict their exact number. A specifically designed filter (W3 code available on GitHub^(^ [Fn fn8] ^)^) accounts for this and also excluding Σ-Ts lacking physical meaning (such as those where not all steps are directed to the designated pond), sensibly reduces the number of spanning trees of interest, which actually becomes 62.[Fn fn5] Table S2 summarizes the identified Σ-T graphs, while Figure 3 illustrates two representations of the first Σ-T graph from that table.

**Figure 3:**
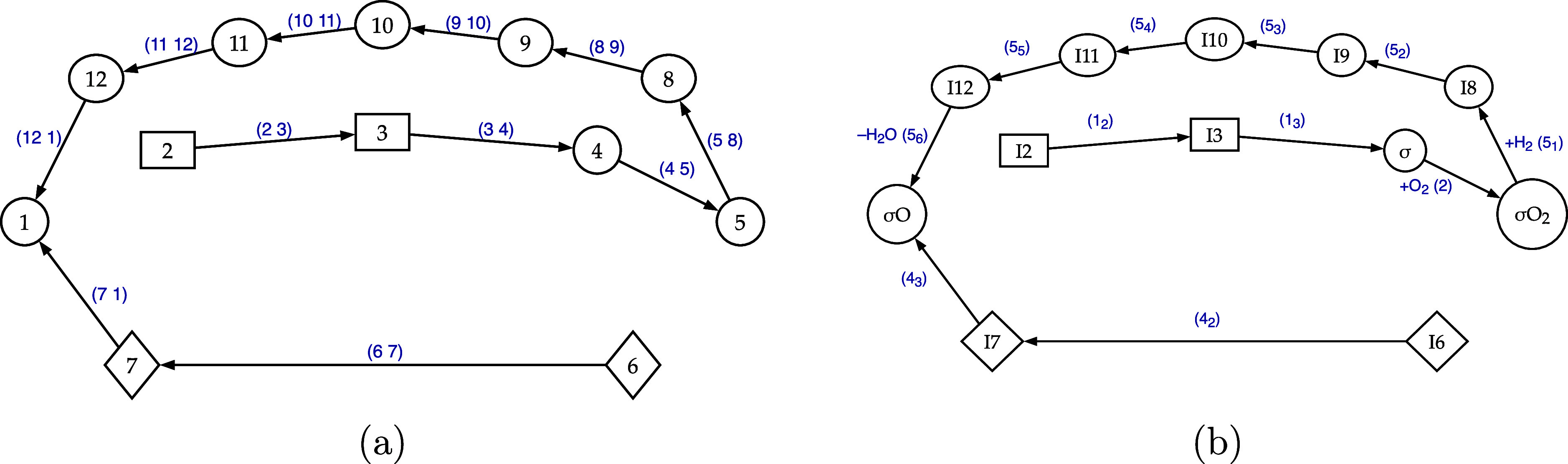
An exemplifying Σ-T, ending on node 1, which concerns the reduced PMK reaction path of the simulated CO/H_2_ PROX on MnO_2_ dispersed catalysts. Graphs (a) and (b) are equivalent, differing only in their notation for nodes/reactants and edges/reactions.

Twelve of these Σ-Ts, for example, have pond on the species σO. This fact can be interpreted by revisiting the waterway metaphor and imagining that the species σO is alimented by the pathways represented by the 12 Σ-T graphs above. In general, the weights of the different Σ-T graphs can be determined as the probabilities of their existence and are thus related to the product of the corresponding event probabilities listed in Table S1. It can be also used the product of the related rates of the molecular events involved, which can be derived from the event probabilities, using eqs 2 and 3.

This is the case, as an example, when the turnover frequency (TOF)^56^ values, useful to characterize the catalyst activity, have to be calculated. Following this line, referring to eqs 2 and 3, and introducing the already discussed approximations on these, the general expression for *D*_*j*_ can be derived

7 $$D_{j}={\left(\frac{k_{B}T}{h}\right)}^{q}{\left[\exp\left(-\frac{\sum_{i}^{m}\Delta G_{i}^{⧧}+\sum_{\gamma }^{n}\Delta G_{\gamma }^{⧧}}{RT}\right)\right]}_{j}$$

Equation 7 is clearly the product of terms from eqs 2 and 3, *j* individuates a generic Σ-T, *q* = *m* + *n* is the total number of steps in the same Σ-T, with *n* and *m* the number of adsorption steps and nonadsorption steps, respectively. The other terms have their standard meaning. In the Σ-T of Figure 3, *q* = 11, while *n* = 1 identifies the step (5 8)/(5_1_) and *m* = 10 includes all other steps.

The pre-exponential term, which seems computationally troubling even for small *q*, is not anyway problematic. This is because the *D*_*j*_ terms are processed as fractions based on ratios of different *D*_*i*_ or other subgraph weight factors, leaving a low value for the exponent (typically 0 or 1) of the $\left(\frac{k_{B}T}{h}\right)$ term that characterizes the pre-exponential factor.

Employing the probabilities instead of the rates, one can operatively consider the sum of the free activation energies, Δ*G* ^⧧^, characterizing the various steps involved in a given Σ-T, which is then used, with its sign reversed and divided by *RT*, as the kernel of the natural exponential function, which collectively fixes the whole probability of the Σ-T graph. Incidentally, it is here recalled that the sum of the Δ*G* ^⧧^ terms has already been introduced in the literature with the acronym TEC, standing for total energy content,^8,57^ and has been used to assess the heuristic relevance of a mechanism among various possible ones.

To return to the context, it should be noted that the weight of a given Σ-T graph is one of the rate-retardant terms *D*_*j*_ either in eqs 5 or 6. At the same time, the summation in the denominator of these equations is extended to include all the Σ-Ts that originate from the graph of the overall reaction pathway, in the present case 62.

Reflecting on the consequences of the metaphor it becomes clear why the sum of the weights, that is, the sum of the probability of existence, of the 12 Σ-Ts leading to the formation of the species σO, normalized with respect to the weights of all the 62 Σ-Ts regarding the formation of all the surface species, including σO, can represent the surface molar ratio of the same σO. Furthermore, it is also evident why the surface fraction, θ_ss_, of any surface species, ss, can be obtained through the following equation^10,27^

8 $$\theta_{ss}=\frac{\sum_{i}D_{i}^{ss}}{\sum_{j}D_{j}}$$

where *D*_*i*_^ss^ and *D*_*j*_, as fixed by eq 7, singly are the products of the weights of the Σ-T graphs related to the given surface species ss, and all the surface species, respectively. Specifically, considering the reference pond as the node corresponding to species σO, i.e., node 1, the subscripts *i* and *j* run, in the order, from 1 to 12 (the tuples showing convergence on node 1) and from 1 to 62 (all the tuples characterizing the overall mechanism), as shown in Table S2.

### X-T Graphs: Coupling Parameters and Branched Cycles

To present the characteristics of the coupling parameters related to the branches of the different cycles, the title system will be initially used for illustration. Generalization is then given at the end of the section. The cycles present in the reaction path of Figure 2, as already stressed, are two: C_2_ and C_3_. In the latter, as shown by the chemical [Disp-formula fdstep_2], one molecule of CO_2_ and one of H_2_O are formed. In the cycle, under pseudosteady-state conditions, there is a substantial equilibrium of the surface species. However, this equilibrium depends on the lateral branches, constituting the X-T graphs. These are made up of steps belonging to the other cycle, C_2_, when they converge on C_3_. The steps above come into contact directly or indirectly with the latter, through the nodes common to the two cycles, i.e., σO and σO_2_, matching the “inlet-nodes” 1 and 5. The lateral branches influencing a given cycle cannot themselves be part of a cycle.^10,27^ Furthermore, to “feed” a given cycle, the lateral branches must be characterized by edges that are either incoming or at least directed to that cycle. From this and the formal algebraic treatment,^10,25,27^ it follows that for cycle C_3_, the lateral branches are composed of the pair of edges ((6 5) (7 1)) and ((6 7) (7 1)). These with cycle C_3_ originate the branched cycle graphs in Figure 4.

**Figure 4:**
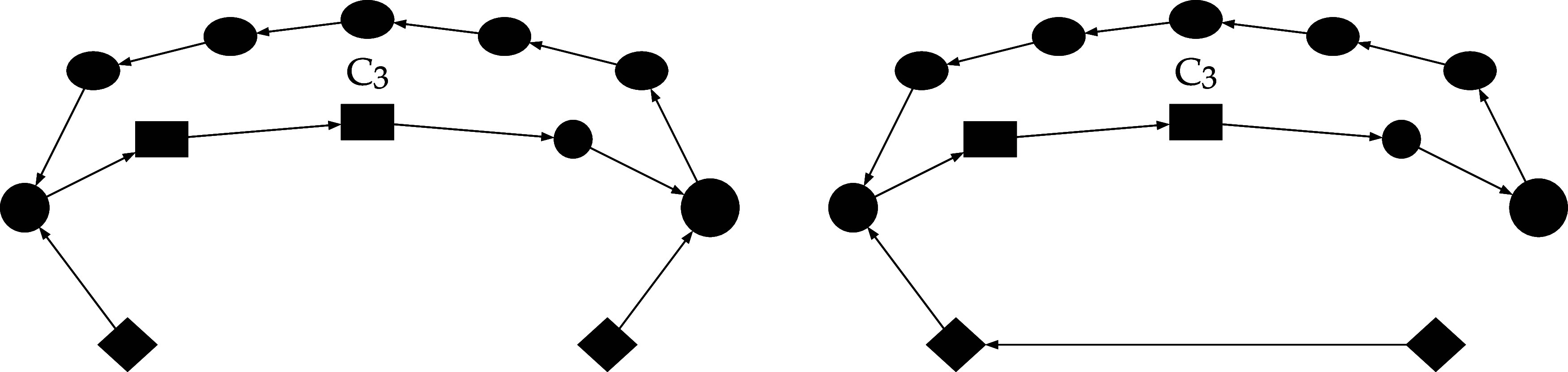
C_3_ branched cycles: the coupling trees, not belonging but touching the cycle, can be easily identified in both the graphs. Reversible steps have been simplified by removing the redundant direction.

Similarly, it is found that cycle C_2_ is connected to four X-T graphs, each comprising five edges, as listed below

$$\begin{array}{l} \left(\left(10\;9\right)\left(9\;8\right)\left(8\;5\right)\left(11\;12\right)\left(12\;1\right)\right);\left(\left(9\;8\right)\left(8\;5\right)\left(10\;11\right)\left(11\;12\right)\left(12\;1\right)\right)\\ \left(\left(8\;5\right)\left(9\;10\right)\left(10\;11\right)\left(11\;12\right)\left(12\;1\right)\right);\left(\left(8\;9\right)\left(9\;10\right)\left(10\;11\right)\left(11\;12\right)\left(12\;1\right)\right) \end{array}$$

which also considering cycle C_2_ correspond, in the order, to the branched cycles in Figure 5.

**Figure 5:**
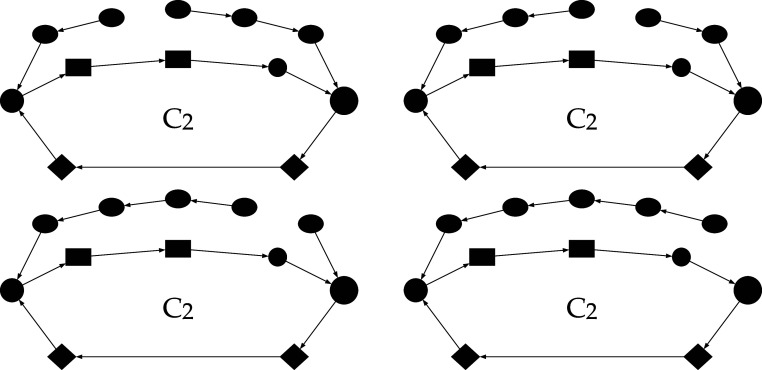
C_2_ branched cycles: the coupling trees, not belonging but touching the cycle, can be easily identified in the graphs. Reversible steps have been simplified by removing the redundant direction.

In the branched cycles above, given the previously mentioned irreversibility of cycles C_2_ and C_3_, only the direct steps following the natural course of the cycle are considered—i.e., the course following the sequence starting from the surface intermediate σO (node 1) and proceeding without including pairs of converging steps/edges at any surface-intermediate/node. As a consequence, in Figures 4 and 5, the reversible steps of the branched cycles have been simplified by removing the redundant direction. For comparison, refer to Figures 4, 5 and 2. The weight of a given *i*-th branched cycle (*C*_[s]*i*_α_*i*_)—either derivable from eq 5 or referable to reaction systems, like the present ones, for which *n* = 1 in the same equation—can be expressed as

9 $$C_{\left[s\right]i}\alpha_{i}={\left[{\left(\frac{k_{B}T}{h}\right)}^{\rho }\exp\left(-\frac{\sum_{c}^{\mu }\Delta G_{c}^{⧧}}{RT}\right)\sum_{z}^{\zeta }\exp\left(-\frac{\sum_{b}^{\upsilon }\Delta G_{b,z}^{⧧}}{RT}\right)\right]}_{i}$$

where *C*_[s]*i*_ as usual is the weight of the considered cycle, and α_*i*_, is the coupling parameter corresponding to the set of related branches.[Fn fn6] The exponent ρ = μ + ν represents the total number of steps in the branched cycle, with μ denoting the number of steps in the cycle and ν the corresponding number of steps in the branches connected to it. It should be noted that, for a given reaction system, the relation ρ = *q* + 1 always holds, where *q* is the equivalent exponent in eq 7. The relation between ρ and *q* actually demonstrates the dimensional consistency of eq 5 and of the activity parameter TOF [s^–1^], which will be employed in the Results and Discussion section. Δ*G*_*c*_^⧧^ and Δ*G*_*b*,*z*_^⧧^ represent the free activation energies of the surface events involved in the catalytic cycle and in the *z*-th branch set, respectively. The aforementioned free activation energy parameters, both contain terms of the type Δ*G*_*i*_^⧧^ and/or Δ*G*_γ_^⧧^ (see eq 7), depending on the absence or presence of adsorptive events, in the corresponding cases. Furthermore, ζ denotes the number of branch groups—each comprising one or two branches—that collectively form the *z*-th set, with each group individually connected to the same cycle. For instance, in the present case, C_2_ features a set of four branch groups—three with two branches and one with a single branch (see Figure 5)—whereas C_3_ has two groups, one with one branch and the other with two (see Figure 4). Clearly, when ζ = 1, the system corresponds to a single branched cycle. Under these conditions, and given the nature of the Δ*G* ^⧧^ terms previously discussed, eq 9—aside from the specific values of ρ and *q*—reduces to a form mathematically equivalent to eq 7.

The following summarizes a more comprehensive analysis on a basic filter algorithm to isolate X-T graphs—associated with the coupling parameters, α_*i*_—obtained from the branches of a cycle originating from a generic polycyclic system. Starting from the polycyclic system, a single cycle is isolated, then the procedure is repeated to isolate all cycles. For each of this cycle, the removed cycles singly generate branches from which (following the protocol described below) significant X-Ts are extracted. These are thus reconnected to the original nodes of the selected cycle, in this way forming the different arms of the branched cycles. Specifically, after selecting one cycle of the polycyclic system as the reference, the arms originating from the set of complementary cycles connected to it are extracted, thereby generating the corresponding branches. This process is then repeated for all separable reference cycles and their complementary ones. Taking one of the complementary cycles as an example, if it contains *n* reversible steps, it may include up to 2^*n*^ independent X-T branches attributable to it.

In order to get significant X-T graphs, the protocol embedded into the W0 code, implementing the filter algorithm available on GitHub^(^ [Fn fn8] ^)^, generates directed branch graphs without symmetric edges and removes those that do not converge on at least one of their originating nodes (linking-nodes) or that diverge from any of these nodes. It further eliminates branch graphs that leave steps or sequences of steps isolated and unconnected to the linking-nodes of the cycle, as well as those lacking directional coherence (i.e., not coherently oriented) toward these nodes. Finally, it removes X-T graphs that exhibit nodes with either out-degree or in-degree ≥ 2. The procedure above should be repeated for all the complementary cycles of any isolable reference cycle in the polycyclic system. This final iterative routine is not yet operational in the W0 code.

### GCODE

*GCODE*—which implements the DFT-GKA approach—was developed in Common Lisp to efficiently handle potentially complex lists of tuples and if needed natively manage very small numbers with arbitrary precision. Specifically, it operates in two stages to identify and use Σ-Ts and X-Ts. Per points, *GCODE*:

(I) To determine Σ-T graphs and the relative energetics:
  (i) Reads the list of tuples containing information about the species (nodes of the graph) and their transformation steps (edges of the graph), and shapes all the possible graphs obtained by removing a number of edges equal to that of the independent cycles—two in the case discussed in this article—eliminating both duplicate graphs and those that still contain cycles after the first filtering;
  (ii) Derives all directed graphs without cycles that do not have any symmetric edges, effectively doubling the number of graphs for each symmetric edge;
  (iii) Identifies all significant Σ-Ts, specifically those that include only steps converging on, or oriented toward, a single node-pond, as illustrated in Figure 3;
  (iv) Evaluating the Δ*G* ^⧧^ values for all the molecular events and considering the partial pressures of the involved species at a given temperature, calculates the weight of each Σ-T that is, calculates using eqs 2 and 3 any *D*_*i*_^ss^ terms and from these all the *D*_*j*_ values and their sum.
(II) To determine X-T graphs and the relative energetics:
  (i) Analyzes individual independent cycles derived from an initial polycyclic system. For each cycle, it identifies sets of appropriately oriented branches associated with the cycles originally connected to the analyzed cycle. These branches, corresponding to the X-T graphs, are then reconnected to the inlet nodes where the originating cycles were cut. This process generates the branched graphs;
  (ii) Considering the Δ*G* ^⧧^ values for all the molecular events and the partial pressures of the involved species at a given temperature, calculates the weights of the different independent cycles (which include specific steps) and their associated X-T coupling graphs. These weights correspond to the terms *C*_[s]*i*_ and α_*i*_, which characterize the branched cycles (see, e.g., Figures 4 and 5) hence sums all contributions, shaping the term ∑_*i*_^*n*^ *C*_[s]*i*_α_*i*_ in the numerator of eq 5 for the relevant steps, s. It is worth recalling that, in the present case, *n* = 1 for each of the involved branched cycles.

All module codes used by *GCODE* are available on GitHub^(^ [Fn fn8] ^)^. In the first phase, points (i)–(iii) and point (iv) are handled by W1–W3 and F1–F3 modules, respectively. In the second phase point (i) and (ii) are sequentially handled by W0 and F1–F3 modules.

## Results and Discussion

As will be shown, the developed machinery characterizing the here introduced DFT-GKA approach enables the extraction of key information on activity, selectivity, surface population, apparent activation energy, and reaction order with respect to the various gas-phase species involved. From eq 5 it is possible to get the rate of formation of CO_2_ and H_2_O hence to obtain the activity and selectivity values at any experimental conditions. For the formation of CO_2_, both C_2_ and C_3_ cycles were used, whereas for H_2_O, only the C_3_ cycle was employed. In both cases, the weights of the steps were determined through eqs 2 and 3, depending on the presence of adsorption events or not. Selectivity—defined as the ratio of the formation rate of a species normalized to the sum of the rates of all formed species—is, in particular, an important parameter in evaluating PROX processes.

Considering the selectivity to H_2_O under moderate temperature conditions, where the SMK routes are either not activated or less significant, the selectivity at the PMK regime $\left(S_{H_{2}O}^{PMK}\right)$ can be determined using the following equation. This equation incorporates the considerations outlined above, particularly the stoichiometry of the chemical [Disp-formula fdstep_1] and [Disp-formula fdstep_2], which governs the overall catalytic reaction

10 $$S_{H_{2}O}^{PMK}=\frac{C_{3}\alpha_{3}}{2C_{3}\alpha_{3}+2C_{2}\alpha_{2}}=0.5\left(\frac{1}{1+\frac{C_{2}\alpha_{2}}{C_{3}\alpha_{3}}}\right)$$

The terms α_2_ and α_3_ are the coupling parameters associated with C_2_ and C_3_ cycles, respectively. Equation 10 can be used to obtain the complementary selectivity to CO_2_: $S_{{CO}_{2}}^{PMK}=1-S_{H_{2}O}^{PMK}$ . It should be noted that in eq 10, the normalization terms ∑_j_ *D*_*j*_, derived from eq 5, cancels out, while C_2_ and C_3_ represent the weights of the cycles, which, as previously mentioned, are denoted in the text using the same symbols (see Figures 4 and 5).

Figure 6 compares the calculated activity-selectivity data for the model Mn_4_O_8_ cluster at various temperatures under typical CO PROX conditions with experimental results obtained for the MnO_2_–CeO_2_ composite catalyst under the same conditions.^3–5^

**Figure 6:**
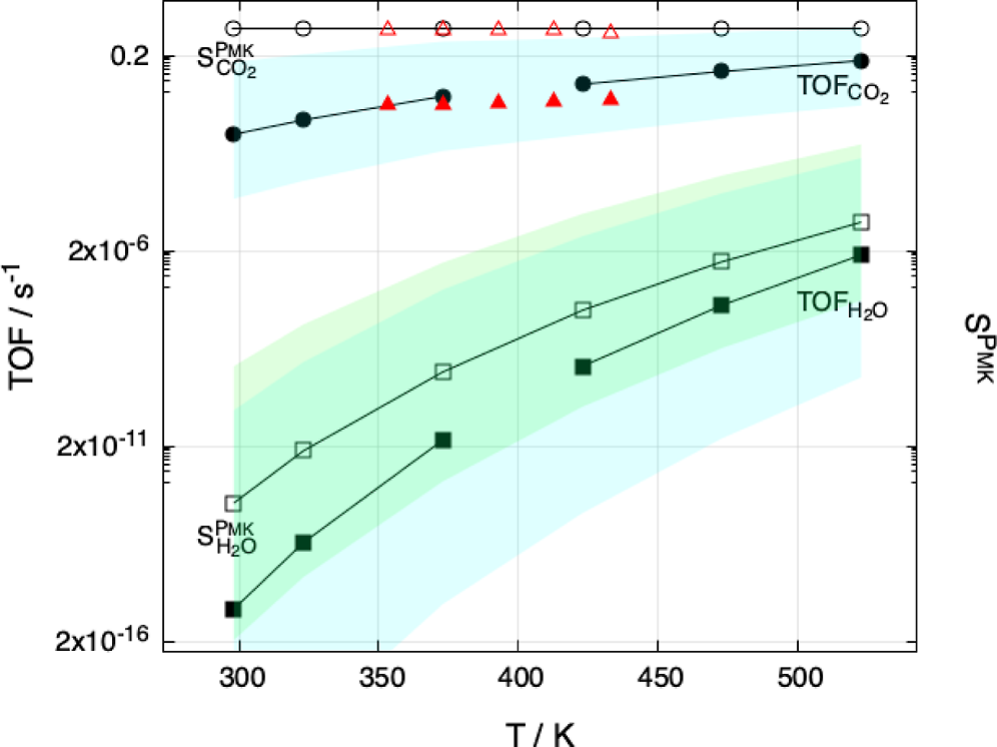
Simulated activity (TOF_*x*_) and selectivity (*S*_*x*_^PMK^) behaviors characterizing both the formation of CO_2_ (filled and empty circles) and H_2_O (filled and empty squares) and the corresponding experimental ${TOF}_{{CO}_{2}}^{\exp}$ and $S_{{CO}_{2}}^{\exp}$ (filled and empty triangles, in red), on MnO_2_–CeO_2_ composite catalysts^5^ at different temperatures. Splines are used to connect the simulated points. The colored bands, cyan and green, represent the sequence of the calculated error bars associated, in the order, with the mimicked TOF_*x*_ and *S*_*x*_^PMK^ descriptors.

The experimental selectivity to CO_2_ ( $S_{{CO}_{2}}^{\exp}$ ) in agreement with eq 10 was determined as $S_{{CO}_{2}}^{\exp}={TOF}_{{CO}_{2}}^{\exp}/\left({TOF}_{{CO}_{2}}^{\exp}+{TOF}_{H_{2}O}^{\exp}\right)$ , being the TOF_*x*_^exp^ terms the experimental activity descriptors of the given *x* species. As a consequence for the present case, it is also valid: $S_{H_{2}O}^{\exp}=1-S_{{CO}_{2}}^{\exp}$ . Recalling that TOF_*x*_/s^–1^ is defined as the number of molecules of *x* produced per time unit on one catalytic site,^56^ simulated TOF_*x*_ values for the different products, namely TOF_CO_2__ and ${TOF}_{H_{2}O}$ , were determined by using eq 5—considering the weights of the branched C_2_ and C_3_ cycles and of the branched C_3_ cycle in the first and second case, respectively—with the corresponding results normalized to four. This is the number of manganese centers in the model cluster considered. The simulated activity and selectivity values reported in Figure 6 were calculated at the partial pressure for CO, O_2_ and H_2_ equal to 1 kPa, for the first two, and 98 kPa, for the latter. The π_ev_ values from Table S1 and eqs 2 and 3 have been used. Experimental and calculated results mostly show good agreement. Error bars associated with the mimicked TOF_*x*_ and *S*_*x*_^PMK^—with *x* ≡ CO_2_, H_2_O—descriptors were also evaluated, incorporating an estimated uncertainty of ±10 kJ mol^–1^ in the 6 free energy barriers characterizing just the transition states^38^ present in the whole process and recalculating the corresponding descriptors.

The evaluation, thus, accounted for cross-checking and comparing all the possible combinations—2^6^ cases for each temperature value[Fn fn7]—of the adjusted free energy barriers. The relatively large error bars that characterize the selectivity and activity parameters—except for the $S_{{CO}_{2}}^{PMK}$ one—are likely due to the error associated with the energy barriers, which is amplified by exponential kernels. Conversely, the very low value that makes the error bars for $S_{{CO}_{2}}^{PMK}$ nearly invisible stems from the definition of selectivity itself. In this case, since the formation rate of H_2_O is negligible compared to that of CO_2_, the selectivity reduces to a ratio of two nearly identical terms. This results in the cancellation of the different error contributions, owing to the exponential nature of the terms involved.

Mimicked selectivities align with the experimental findings, which show that water selectivity remains quite low but slightly increases with temperature. With respect to this, it is interesting to observe that the selectivity has a growth rate that decreases as the temperature increases and as the molar fraction of the σO_2_ sites increases. This fraction however grows slowly within the temperature range considered, from 0.00@298 K to a value of 0.02@523 K. The σO sites are the only other surface species significantly present and they actually show the complementary molar fraction values with respect to the σO_2_ sites at equilibrium. Consistently with the increase in σO_2_, while the selectivity to CO_2_ remains almost constant, it can be observed that the selectivity to H_2_O follows a trend similar to that of the ${TOF}_{H_{2}O}$ . In particular, if the trends of the first and last three ${TOF}_{H_{2}O}$ points of Figure 6 are considered as separate linear trends, it can be observed that the ratio of the slopes between the first and last segments is approximately 4 × 10^5^. This can be compared to the analogous ratio for TOF_CO_2__ over the same intervals, which is equal to 5. This clearly demonstrates that the oxidation of H_2_, compared to that of CO, is more sensitive either to the temperature variations or to the increase of the hyper-oxygenated σO_2_ species on the surface.

Referring to the simulated results, the apparent activation energy, *E*_a_^app^,^58^ can be determined using the modified version of the Arrhenius equation,^56^ as reported below^58^

11 $$E_{a}^{app}=-R\left[\frac{\partial \log\left(r\right)}{\partial \left(1/T\right)}\right]$$

where *r* is the reaction rate, simultaneously considering the formation of the products, here CO_2_ and H_2_O. Since one can formally write TOF = *r*/ν_σ_, where ν_σ_ is the number of available catalytic sites, it is clear that in eq 11, *r* can be replaced by TOF.^40^ In this case, TOF_CO_2__ plus ${TOF}_{H_{2}O}$ .

In the two temperature ranges considered—namely, 298–373 and 423–523 K—the *E*_a_^app^ values were found to be 27.(6) kJ mol^–1^ at lower temperatures and 25.(5) kJ mol^–1^ at higher temperatures. The observed 8% variation between the two *E*_a_^app^ values, combined with an asymptotic standard error less than 2% in the determination of *E*_a_^app^—which would reflect in a ±0.5 kJ mol^–1^ uncertainty in the calculated values—allows us to confidently infer that the two apparent energy barriers mirror distinct trends. The maintenance, or even the increase, of this percentage variation observed by recalculating the apparent activation energy values from the trends of the values taken at the upper and lower extremes of the error band related to the TOF_CO_2__, confirm the soundness of the previous observation. In any case, this change appears to suggest some influence of the increase in the σO_2_ sites on the reaction mechanism.

The results so far presented, as a whole, indicate that the two oxidation processes characterizing CO and H_2_, particularly at lower temperatures, occur on different time scales and therefore almost independently. This independence thus would justify the foundations of the macro-kinetic experimental study presented in refs 3–5 , which treats the two oxidation processes as effectively unrelated. However, the increase in the mole fraction of hyper-oxygenated sites, apparently activated by a rise in temperature, brings the rates of the two processes closer together. Moreover, the inclusion of steps introducing the SMK mechanisms into the reaction process at higher temperatures, particularly step (6) in Figure 1, will certainly increase the presence of σO_2_ sites. The increase in these sites, as observed until now, should favor the production of H_2_O over CO_2_, in this way increasing water selectivity as experimentally observed at higher temperatures.

The apparent reaction orders for the different species at the two temperatures considered (373 and 523 K) were calculated using the expression: $TOF={kp}_{CO}^{l}p_{H_{2}}^{m}p_{O_{2}}^{n}p_{N_{2}}^{0}$ , where TOF may refer to the production of either CO_2_, H_2_O or both, *k* is the rate constant and *p*_*x*_ the partial pressure of the generic species X, while *l*, *m*, *n*, and zero are the reaction orders of the different species, here CO, H_2_, O_2_, N_2_. The activity expression above, characterized by the absence of CO_2_ and H_2_O and involving an apparent zero-order dependence on N_2_ (introduced as a diluent), arises because molecular nitrogen does not directly participate in the reaction mechanism, while the contributions of carbon dioxide and water produced during the reaction are negligible to the overall process dynamics.

To determine the reaction order for each species X with respect to the production of water, carbon dioxide or both it is thus possible to consider two different partial pressure values, *p*_*x*1_ and *p*_*x*2_ = *n*·*p*_*x*1_, at a given temperature, while keeping the partial pressures of all other species constant. In fact, if the reaction order for this species is *y*_*x*_, it can be easily derived the reaction order with respect to X by the following equation

$$y_{x}=\log_{n}\frac{{TOF}_{2}}{{TOF}_{1}}$$

being *n* = *p*_*x*2_/*p*_*x*1_ the basis of the logarithm while TOF_2_ and TOF_1_ the turnover frequency values referred to the production of some species when the partial pressures of X are equal to *p*_*x*2_ and *p*_*x*1_, respectively. Table 3 reports the reaction orders for the different species at the here considered temperatures and partial pressures of the components of the gas mixtures, summarized in the table notes. Using the previously described procedure to correct for the effects of the partial pressures of the gas-phase components, the reaction orders of the different reagents involved in the PROX reaction, with respect to the formation of CO_2_ and of the simultaneous formation of CO_2_ and H_2_O, confirmed that the oxidation of CO could be considered almost independent of that of H_2_. Furthermore, the formation of CO_2_ appears to be linearly influenced by the partial pressure of CO. However, as the temperature increases, there is a slight decrease of this effect. At low temperatures, CO does not affect the formation of H_2_O, but unexpectedly, it seems to become significant for its formation at higher temperatures.

**Table 3:** Simulated Reaction Order (**r.o.**) Values for the PROX Reaction on the Mn_4_O_8_ Fragment: Formation of CO_2_, H_2_O and Combined Products, for CO (**y**_**CO**_), O_2_ (**y**_O_2__), and H_2_ (**y**_H_2__) at Two Different Temperatures

	formed species^a^		
**r.o.** ^b^	CO_2_	H_2_O	CO_2_ + H_2_O
**y**_**CO**_	1.00|0.95	0.00|1.12	1.00|0.95
**y**_H_2__	–0.66|−0.65	0.34|0.34	–0.66|−0.64
**y**_O_2__	0.00|0.00	0.00|0.00	0.00|0.00

a The vertical bar distinguishes the reaction order values at 373 on the left and 523 K on the right, respectively.

b The reaction orders were obtained by cross-referencing the effects on reactivity at the two temperatures considered for different gas mixtures containing CO, O_2_, H_2_, and N_2_, at a total pressure of 100 kPa, with the proportions indicated by the following tuples: (1 1 98 0), (1 1 49 49), (5 1 49 45), and (1 5 49 45).

Likely, this could reflect the intrinsic limits of the model approximations, which tend to lose accuracy at higher temperatures. An alternative interpretation would suggest that increasing the temperature favors steps (1) and (2), leading to the formation of the σO_2_ site, which in turn, as observed, causes a shift in the selectivity toward H_2_O. In this case, this inference would provide further evidence that the increase in the number of σO_2_ sites induces changes in the reaction mechanism, favoring the formation of H_2_O. Hydrogen seems conversely to have a negative effect on the formation of CO_2_ and a foregone positive one on H_2_O. The former effect is negligible, especially at lower temperatures, given the extremely high selectivity to CO_2_ observed. The most remarkable result, however, concerns the pseudozero order that characterizes the role of molecular oxygen. This finding, along with the experimental data,^5^ highlights that the oxidation is primarily driven by structural (lattice) oxygen present in the MnO_2_ dispersed fragments, modeled by the Mn_4_O_8_ units.^3–5^ The oxygen of the deoxygenated fragments would only be restored afterward by molecular oxygen.

Given the analysis above, it could finally be stated that the present study may be considered exhaustive for the CO PROX process on the Mn_4_O_8_ fragment catalysts in the lower temperature range (298–373 K), where the PMK processes were identified. For the higher temperature range, where SMK processes should ideally be activated, further investigation is required. This, in addition to considering steps (3) and (6) of Figure 1, also would require a DFT study of the diffusion processes of surface oxygen species on an effective MnO_2_–CeO_2_ composite catalyst model, using larger and potentially periodic catalytic systems.

A final comment concerns the DFT data used and the associated estimated errors in evaluating the free energy barriers of the transition states involved in the whole reaction mechanism. While it is clear that reducing these errors would increase confidence in the results, it is equally evident that this aspect does not seem to affect the conclusions of the present work.

## Conclusions

Density functional theory-based graph kinetic analysis (DFT-GKA) of the CO preferential oxidation in H_2_ rich feedstocks (PROX) on a Mn_4_O_8_ cluster, modeling a MnCeO_*x*_ nanocomposite catalyst, has validated earlier experimental and computational results. The study establishes a foundational framework for deeper mechanistic investigations into the title reaction. Specifically, DFT-GKA method overcomes the need to separately model the two oxidation processes to study the CO/H_2_ PROX on MnO_2_–CeO_2_ composite catalysts. Additionally, it demonstrates the reliability of this approximation in lower temperature regimes. On the whole, it also gives a more comprehensive overview on the activity and selectivity descriptors of the investigated process, ultimately proving to be a novel, potentially robust and effective analytical tool for studying complex heterogeneous catalytic processes, particularly midscale ones, like the one illustrated.

## Supplementary Material



## Footnotes

a It has here to be recalled that the rate constants as expressed by Eqs 2 and 3, are normalized to the fraction of the corresponding surface sites occupied. This is in agreement with the perspective of an observer point of view focused on just one catalytically active surface unit.

b While noncanonical (parasitic) processes are generally less studied, they are critical in depositing residual species on the catalyst surface. These processes induce aging phenomena, which can significantly modify both the catalyst and its catalytic activity—either through poisoning or promotion. Collectively, these residual species could be referred to as *alter* species.^52^

c Hereafter, any term such as *C*_[s]_, *C*_[s]*i*_, or any other notation representing a cycle will denote both the cycle itself and the product of the rates of its constituent steps.

d Once upon a time, in Arab-Norman Palermo, there was a fabulous garden known as Genoardo. This garden had a complex system of springs, channels, wells, and ponds. Ponds often collected water in lush pleasant places where people used to talk about art, philosophy and, we like to think, the deepest essence of algebra. The *homemade* Common Lisp *GCODE* is employed in this work to derive and use the Σ-Ts and X-Ts. *G* in the acronym of the code want to recall this garden.

e The average run-time to obtain the final 62 Σ-Ts, was on the order of 10^–2^ s, running a 3-module interpreted Lisp code (W1, W2, W3@https://github.com/enonmorferehwon/gcode) in a SBCL environment on a MacBook Pro equipped with a 2.3 GHz Intel Core i7 quad-core processor and 32 GB of RAM (3733 MHz LPDDR4X), under Sequoia 15.3.

f It must always be stressed that the term α_*i*_ corresponds to a summation that includes the weights of the individual X-T graphs conjugated to a given cycle *C*_[s]*i*_, each multiplied by the weight of the latter.^10^ For this, a more formal expression for the numerator of eq 5 could be ∑_*i*_^*n*^∑_*k*_ *C*_[s]*i*_α_*ik*_.

g The average run-time to obtain the whole kinetic results concerning the final 2^12^ examined systems, lasted less than 1 min running the 3-module interpreted Lisp code (F1, F2, F3@https://github.com/enonmorferehwon/gcode) in a SBCL environment on a Dell XPS Notebook equipped with Intel Core i7-1250U processor and 16 GB of LPDDR5 RAM at 5200 MHz, under Pop!_OS 22.04 LTS.

h Structural and energetic data for the various species involved in the catalytic mechanism were obtained employing the Gaussian 09 suite of programs, which, while not free, is available under an academic license.^59^ Detailed structural data, along with absolute energetic (ZPVE) information for all catalytic species and transition states in the cyclic paths, are provided in a freely accessible Supporting Information file. In the latter, the numbering of the involved species are in agreement with that already used in ref 3. From the ZPVE data, the free-energy (Δ*G* ^⧧^) related results, fundamental in this study, can be derived using the Goodvibes code,^60^ as detailed in the Computational Details section.^37^ Supporting Information also reports information on the kinetics of the title process and on a parallel equilibrium process, involving the CeO_2_ matrix of the MnO_2_–CeO_2_ composite catalyst modeled. The *GCODE* implementing the DFT-GKA algorithm is fully described in dedicated sections of the work in addition to the equations it employs. At the address https://github.com/enonmorferehwon/gcode, with a readme file (README.pdf), an example is provided to extract information in complex catalytic cycles by using *GCODE*. This is mainly addressed to the analysis of the PROX catalytic system at *T* = 298 K and *p*_tot_ = 100 kPa, for CO, H_2_, O_2_, and N_2_ percentages of 1, 1, 98, and 0, respectively. However, the instructions for the necessary modifications to the code as well as input files to reproduce and extend the results reported in the article are also included.

## Notes

The file ci5c00072_SI.pdf, containing structural and kinetic information pertaining to the proposed DFT-GKA approach for the title reaction (PDF)
