# Supplementary material for: Alternative Algebraic Perspectives on CO/H2 PROX over MnO2 Composite Catalysts
Source: J Chem Inf Model. 2025 May 2;65(10):4952–67. doi: 10.1021/acs.jcim.5c00072 (PMC12117570; doi:10.1021/acs.jcim.5c00072)
Supplement: Supplementary file 1 [file ci5c00072_si_001.pdf]

Supporting Information for

Alternative Algebraic Perspectives on CO/H_2 PROX over MnO_2 Composite Catalysts

Marco Bertini,[†] Francesco Ferrante,[†] Laura Gueci,[†] Antonio Prestianni,[†] Dario Duca,[*,†] Francesco Arena,[‡] and Dmitry Yu. Murzin[¶]

†*Dipartimento di Fisica e Chimica "Emilio Segrè", Università degli Studi di Palermo, Viale delle Scienze Ed. 17, I-90128 Palermo, Italy*

‡*Dipartimento di Ingegneria, Università degli Studi di Messina, Contrada di Dio, I-98166 Messina, Italy*

¶*Laboratory of Industrial Chemistry and Reaction Engineering, Johan Gadolin Process Chemistry Centre, Åbo Akademi University, Henriksgatan 2, 20500 Turku/Åbo, Finland*

E-mail: dario.duca@unipa.it

Phone: +39-091-238-97975. Fax: +39-091-590015

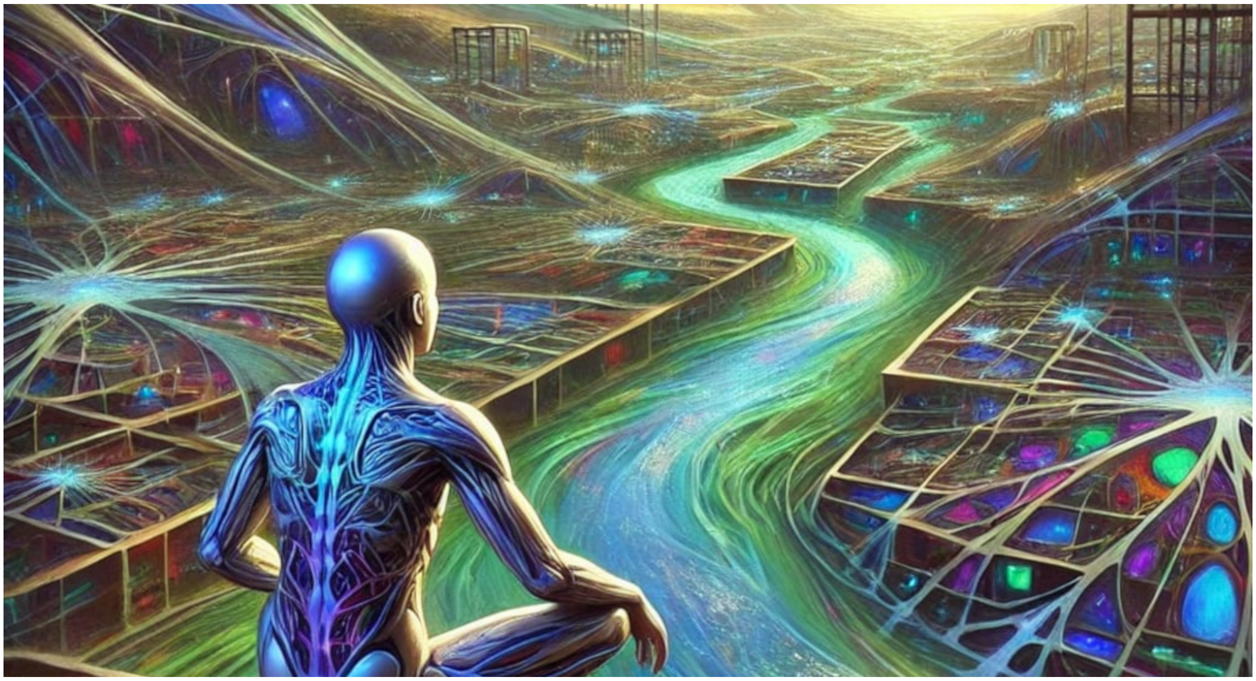

Red references refer to content into this Supporting Information; purple to the Main Text

Table S1: CO/H_2 PROX reaction on manganese oxide fragment: activation energy barrier ($\Delta G^{\ddagger}$) and occurrence probability on a catalytic surface unit (π_{ev}) characterizing the different elementary steps (**S**) at different temperature (**T**)

	**T / K**			
	298		323	
**S**[a]	$\Delta G^{\ddagger}$/kJmol^{-1}	π_{ev}^{b}	$\Delta G^{\ddagger}$/kJmol^{-1}	π_{ev}
(1_1)	16.5 | 0.0	$1.3 \cdot 10^{-3}$ | $1.0 \cdot 10^{0}$	20.2 | 0.0	$5.4 \cdot 10^{-4}$ | $1.0 \cdot 10^{0}$
(1_2)	55.6 | 235.2	$1.8 \cdot 10^{-10}$ | $0.0 \cdot 10^{0}$	56.1 | 235.7	$8.5 \cdot 10^{-10}$ | $0.0 \cdot 10^{0}$
(1_3)	19.4 | ∞	$4.0 \cdot 10^{-4}$ | $0.0 \cdot 10^{0}$	15.6 | ∞	$3.0 \cdot 10^{-3}$ | $0.0 \cdot 10^{0}$
(2)	0.0 | ∞	$1.0 \cdot 10^{0}$ | $0.0 \cdot 10^{0}$	0.0 | ∞	$1.0 \cdot 10^{0}$ | $0.0 \cdot 10^{0}$
(4_1)	4.9 | 0.0	$1.4 \cdot 10^{-1}$ | $1.0 \cdot 10^{0}$	9.1 | 0.0	$3.4 \cdot 10^{-2}$ | $1.0 \cdot 10^{0}$
(4_2)	46.4 | ∞	$7.4 \cdot 10^{-9}$ | $0.0 \cdot 10^{0}$	46.7 | ∞	$2.8 \cdot 10^{-8}$ | $0.0 \cdot 10^{0}$
(4_3)	14.9 | ∞	$2.4 \cdot 10^{-3}$ | $0.0 \cdot 10^{0}$	11.1 | ∞	$1.6 \cdot 10^{-2}$ | $0.0 \cdot 10^{0}$
(5_1)	4.4 | 0.0	$1.7 \cdot 10^{-1}$ | $1.0 \cdot 10^{0}$	6.7 | 0.0	$8.3 \cdot 10^{-2}$ | $1.0 \cdot 10^{0}$
(5_2)	113.5 | 83.7	$0.0 \cdot 10^{0}$ | $2.1 \cdot 10^{-15}$	114.5 | 83.9	$3.0 \cdot 10^{-19}$ | $2.7 \cdot 10^{-14}$
(5_3)	46.8 | 48.6	$6.3 \cdot 10^{-9}$ | $3.0 \cdot 10^{-9}$	46.8 | 48.7	$2.7 \cdot 10^{-8}$ | $1.3 \cdot 10^{-8}$
(5_4)	101.2 | 312.4	$1.8 \cdot 10^{-18}$ | $0.0 \cdot 10^{0}$	101.3 | 312.8	$4.1 \cdot 10^{-17}$ | $0.0 \cdot 10^{0}$
(5_5)	71.9 | 210.4	$2.5 \cdot 10^{-13}$ | $0.0 \cdot 10^{0}$	72.2 | 210.4	$2.1 \cdot 10^{-12}$ | $0.0 \cdot 10^{0}$
(5_6)	31.7 | ∞	$2.8 \cdot 10^{-6}$ | $0.0 \cdot 10^{0}$	28.0 | ∞	$3.0 \cdot 10^{-5}$ | $0.0 \cdot 10^{0}$

	**T / K**			
	373	423	473	523
		π_{ev}		
(1_1)	$1.7 \cdot 10^{-4}$ | $1.0 \cdot 10^{0}$	$4.5 \cdot 10^{-5}$ | $1.0 \cdot 10^{0}$	$1.9 \cdot 10^{-5}$ | $1.0 \cdot 10^{0}$	$9.9 \cdot 10^{-6}$ | $1.0 \cdot 10^{0}$
(1_2)	$1.0 \cdot 10^{-8}$ | $0.0 \cdot 10^{0}$	$6.9 \cdot 10^{-8}$ | $0.0 \cdot 10^{0}$	$3.0 \cdot 10^{-7}$ | $0.0 \cdot 10^{0}$	$1.0 \cdot 10^{-6}$ | $0.0 \cdot 10^{0}$
(1_3)	$1.3 \cdot 10^{-3}$ | $0.0 \cdot 10^{0}$	$2.6 \cdot 10^{-2}$ | $0.0 \cdot 10^{0}$	$2.6 \cdot 10^{-1}$ | $0.0 \cdot 10^{0}$	$1.0 \cdot 10^{0}$ | $0.0 \cdot 10^{0}$
(2)	$1.0 \cdot 10^{0}$ | $0.0 \cdot 10^{0}$	$1.0 \cdot 10^{0}$ | $0.0 \cdot 10^{0}$	$1.0 \cdot 10^{0}$ | $0.0 \cdot 10^{0}$	$1.0 \cdot 10^{0}$ | $0.0 \cdot 10^{0}$
(4_1)	$3.8 \cdot 10^{-3}$ | $1.0 \cdot 10^{0}$	$7.1 \cdot 10^{-4}$ | $1.0 \cdot 10^{0}$	$1.9 \cdot 10^{-4}$ | $1.0 \cdot 10^{0}$	$6.5 \cdot 10^{-5}$ | $1.0 \cdot 10^{0}$
(4_2)	$2.4 \cdot 10^{-7}$ | $0.0 \cdot 10^{0}$	$1.2 \cdot 10^{-6}$ | $0.0 \cdot 10^{0}$	$4.1 \cdot 10^{-6}$ | $0.0 \cdot 10^{0}$	$1.1 \cdot 10^{-5}$ | $0.0 \cdot 10^{0}$
(4_3)	$3.2 \cdot 10^{-1}$ | $0.0 \cdot 10^{0}$	$1.0 \cdot 10^{0}$ | $0.0 \cdot 10^{0}$	$1.0 \cdot 10^{0}$ | $0.0 \cdot 10^{0}$	$1.0 \cdot 10^{0}$ | $0.0 \cdot 10^{0}$
(5_1)	$2.5 \cdot 10^{-2}$ | $1.0 \cdot 10^{0}$	$1.0 \cdot 10^{-2}$ | $1.0 \cdot 10^{0}$	$5.0 \cdot 10^{-3}$ | $1.0 \cdot 10^{0}$	$2.9 \cdot 10^{-3}$ | $1.0 \cdot 10^{0}$
(5_2)	$4.5 \cdot 10^{-17}$ | $1.6 \cdot 10^{-12}$	$2.0 \cdot 10^{-15}$ | $3.3 \cdot 10^{-11}$	$3.9 \cdot 10^{-14}$ | $3.7 \cdot 10^{-10}$	$4.2 \cdot 10^{-13}$ | $2.6 \cdot 10^{-9}$
(5_3)	$2.7 \cdot 10^{-7}$ | $1.5 \cdot 10^{-7}$	$1.6 \cdot 10^{-6}$ | $9.1 \cdot 10^{-7}$	$6.3 \cdot 10^{-6}$ | $3.9 \cdot 10^{-6}$	$1.9 \cdot 10^{-5}$ | $1.2 \cdot 10^{-5}$
(5_4)	$5.9 \cdot 10^{-15}$ | $1.0 \cdot 10^{0}$	$2.6 \cdot 10^{-13}$ | $1.0 \cdot 10^{0}$	$5.2 \cdot 10^{-12}$ | $1.0 \cdot 10^{0}$	$5.8 \cdot 10^{-11}$ | $1.0 \cdot 10^{0}$
(5_5)	$6.6 \cdot 10^{-11}$ | $1.0 \cdot 10^{0}$	$8.6 \cdot 10^{-10}$ | $1.0 \cdot 10^{0}$	$6.6 \cdot 10^{-9}$ | $1.0 \cdot 10^{0}$	$3.3 \cdot 10^{-8}$ | $1.0 \cdot 10^{0}$
(5_6)	$1.3 \cdot 10^{-3}$ | $0.0 \cdot 10^{0}$	$2.3 \cdot 10^{-2}$ | $0.0 \cdot 10^{0}$	$2.2 \cdot 10^{-1}$ | $0.0 \cdot 10^{0}$	$1.0 \cdot 10^{0}$ | $0.0 \cdot 10^{0}$

[a] Excluding spillover effects (dashed lines), **S** is any elementary steps involved in the surface processes, as reported in Figure 1.
[b] π_{ev} values are calculated, for "forward | backward" steps, by the exponential factor of the Eyring-Polanyi equation,[1] using the corresponding $\Delta G^{\ddagger}$ values determined by DFT, applying the Grimme[2] method and using the Goodvibes code.[3] These values for the range of temperatures 373 – 523 K can be found in Ref. 4. Probabilities having values lower than 10^{-20} were fixed to 0. In fact, at the largest temperature here considered, the corresponding event step rate would result *ca.* 10^{-7} / s, which is a very low value for a surface intermediate transforming on a catalytic site. For other details see the related text.

Red-ox processes on the CeO_2 matrix of the MnO_2-CeO_2 composite catalyst

The active sites of the CeO_2 matrix present in the composite MnO_2-CeO_2 catalyst,[4,5] depending on their oxidation state, are denoted as χO, χ, and χO_2, using a notation consistent with that already employed for the manganese oxide fragments discussed in the article.

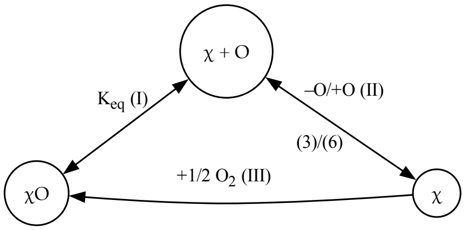

Scheme S1: Simplified cycle representation occurring on the CeO_2 fragments: the nodes in the graph represent the different oxidation states of the Ce sites; equilibrium (I) refers to the process of oxygen vacancy formation $\chi O \rightleftharpoons \chi + O$. Step (II) involves the use of surface atomic oxygen, initiating oxygen spillover to the manganese fragments; it is not excluded the back reaction due to an exchange equilibrium involving oxygen originating by the MnO_2 matrix. Step (III) represents the restoration of oxygenated sites on CeO_2 through molecular oxygen. The notation (3)/(6) on edge (II) highlights that the corresponding step is essential for the occurrence of events (3) and (6) in Scheme 1.

The cycle {I,II,III} in Scheme S1 operates independently of those from Scheme 1, serving as an atomic oxygen buffer that likely becomes active at a specific temperature threshold. Below that temperature the cycles containing steps that involve atomic oxygen transfer, namely steps (3) and (6) of Scheme 1, would conversely become irrelevant to the overall mechanism. Due to the supply of lattice atomic O by the structural CeO_2 promoter, the cycles involving steps (3) and/or (6) were labeled as SMK, that is secondary Mars-van Krevelen, cycles; the remaining ones as PMK, primary Mars-van Krevelen, cycles. Given this, PMK and SMK processes should occur at lower and higher temperature, respectively. Note that the terms *primary* and *secondary* are meant to emphasize the direct involvement in the oxidation processes of lattice oxygens in matrices containing Mn and Ce, in that order. Assuming an explicit mixing of the two surface mechanisms from Schemes 1 and S1, it can be observed that among steps (I), (3), and (6), one combination forms a non-cyclic path, summarized as [+I,+3,−6,−I], resulting in apparent manganese oxide disproportionation. This parallel mechanism was however deemed irrelevant.

Extended tuples individuating the essential kinetic information of a process

Different species, corresponding to nodes of a graph, can be identified by numbers, while elementary events, corresponding to weighted edges of the graph, can be represented as tuples of three numbers. In the tuples, the first two numbers identify, in the order, surface reactants and products, while the third value is related to an activation energy parameter — such as the activation free energy barrier, $\Delta G^{\ddagger}$, between the reactant and the TS preceding the formation of the product — or the related probability of occurrence (per unit of time) of the event involving the same energy parameter.

((1 2 45.34) (2 1 .0000) (2 3 57.00) (3 4 20.50) (4 5 18.58)
(5 6 35.64) (5 8 11.52) (6 5 .0000) (6 7 47.30) (7 1 3.500)
(8 5 .0000) (8 9 116.7) (9 8 84.30) (9 10 46.90) (10 9 48.80)
(10 11 101.6) (11 12 72.70) (12 1 20.60))

Employing the free activation energy parameters originating the occurrence probability values of Tables S1, the mechanism of Figure 2 at 373 K — after having corrected by Eq. 3 the energy barriers of the steps involving adsorption for an initial gas-phase composition implying the mole fractions χ_{CO}, χ_{O_2} and χ_{H_2} equal to 0.01, 0.01 and 0.98, respectively — could be represented by the vector of tuples above.

Table S2: Effective spanning tree (**Σ-T**) graphs and their sequential (***n***) numbers, characterizing the whole reaction path of PROX on MnO_2-CeO_2 composite catalysts, involving lattice oxygens originating mainly from MnO_2 matrices (Pmk processes)

n[a]	**Σ-T**[b]	***n***	**Σ-T**
**1**	((2 3) (3 4) (4 5) (5 8) (6 7) (7 1) (8 9) (9 10) (10 11) (11 12) (12 1))	32	((1 2) (2 3) (3 4) (4 5) (6 5) (7 1) (8 9) (9 10) (10 11) (11 12) (12 1))
2	((2 1) (3 4) (4 5) (5 8) (6 7) (7 1) (8 9) (9 10) (10 11) (11 12) (12 1))	33	((1 2) (2 3) (3 4) (4 5) (6 7) (7 1) (8 5) (9 10) (10 11) (11 12) (12 1))
3	((2 3) (3 4) (4 5) (5 8) (6 5) (7 1) (8 9) (9 10) (10 11) (11 12) (12 1))	34	((1 2) (2 3) (3 4) (4 5) (6 5) (7 1) (8 5) (9 10) (10 11) (11 12) (12 1))
4	((2 1) (3 4) (4 5) (5 8) (6 5) (7 1) (8 9) (9 10) (10 11) (11 12) (12 1))	35	((1 2) (2 3) (3 4) (4 5) (6 7) (7 1) (8 5) (9 8) (10 11) (11 12) (12 1))
5	((2 3) (3 4) (4 5) (5 6) (6 7) (7 1) (8 9) (9 10) (10 11) (11 12) (12 1))	36	((1 2) (2 3) (3 4) (4 5) (6 5) (7 1) (8 5) (9 8) (10 11) (11 12) (12 1))
6	((2 1) (3 4) (4 5) (5 6) (6 7) (7 1) (8 9) (9 10) (10 11) (11 12) (12 1))	37	((1 2) (2 3) (3 4) (4 5) (6 7) (7 1) (8 5) (9 8) (10 9) (11 12) (12 1))
7	((2 3) (3 4) (4 5) (5 6) (6 7) (7 1) (8 5) (9 10) (10 11) (11 12) (12 1))	**38**	((1 2) (2 3) (3 4) (4 5) (6 5) (7 1) (8 5) (9 8) (10 9) (11 12) (12 1))
8	((2 1) (3 4) (4 5) (5 6) (6 7) (7 1) (8 5) (9 10) (10 11) (11 12) (12 1))	**39**	((1 2) (2 3) (3 4) (4 5) (5 6) (7 1) (8 9) (9 10) (10 11) (11 12) (12 1))
9	((2 3) (3 4) (4 5) (5 6) (6 7) (7 1) (8 5) (9 8) (10 11) (11 12) (12 1))	40	((1 2) (2 3) (3 4) (4 5) (5 6) (7 1) (8 5) (9 10) (10 11) (11 12) (12 1))
10	((2 1) (3 4) (4 5) (5 6) (6 7) (7 1) (8 5) (9 8) (10 11) (11 12) (12 1))	41	((1 2) (2 3) (3 4) (4 5) (5 6) (7 1) (8 5) (9 8) (10 11) (11 12) (12 1))
11	((2 3) (3 4) (4 5) (5 6) (6 7) (7 1) (8 5) (9 8) (10 9) (11 12) (12 1))	**42**	((1 2) (2 3) (3 4) (4 5) (5 6) (7 1) (8 5) (9 8) (10 9) (11 12) (12 1))
**12**	((2 1) (3 4) (4 5) (5 6) (6 7) (7 1) (8 5) (9 8) (10 9) (11 12) (12 1))	**43**	((1 2) (2 3) (3 4) (4 5) (5 6) (6 7) (8 9) (9 10) (10 11) (11 12) (12 1))
**13**	((1 2) (3 4) (4 5) (5 8) (6 7) (7 1) (8 9) (9 10) (10 11) (11 12) (12 1))	44	((1 2) (2 3) (3 4) (4 5) (5 6) (6 7) (8 5) (9 10) (10 11) (11 12) (12 1))
14	((1 2) (3 4) (4 5) (5 8) (6 5) (7 1) (8 9) (9 10) (10 11) (11 12) (12 1))	45	((1 2) (2 3) (3 4) (4 5) (5 6) (6 7) (8 5) (9 8) (10 11) (11 12) (12 1))
15	((1 2) (3 4) (4 5) (5 6) (6 7) (7 1) (8 9) (9 10) (10 11) (11 12) (12 1))	**46**	((1 2) (2 3) (3 4) (4 5) (5 6) (6 7) (8 5) (9 8) (10 9) (11 12) (12 1))
16	((1 2) (3 4) (4 5) (5 6) (6 7) (7 1) (8 5) (9 10) (10 11) (11 12) (12 1))	**47**	((1 2) (2 3) (3 4) (4 5) (5 8) (6 7) (7 1) (9 10) (10 11) (11 12) (12 1))
17	((1 2) (3 4) (4 5) (5 6) (6 7) (7 1) (8 5) (9 8) (10 11) (11 12) (12 1))	48	((1 2) (2 3) (3 4) (4 5) (5 8) (6 5) (7 1) (9 10) (10 11) (11 12) (12 1))
**18**	((1 2) (3 4) (4 5) (5 6) (6 7) (7 1) (8 5) (9 8) (10 9) (11 12) (12 1))	49	((1 2) (2 3) (3 4) (4 5) (5 8) (6 7) (7 1) (9 8) (10 11) (11 12) (12 1))
**19**	((1 2) (2 3) (4 5) (5 8) (6 7) (7 1) (8 9) (9 10) (10 11) (11 12) (12 1))	50	((1 2) (2 3) (3 4) (4 5) (5 8) (6 5) (7 1) (9 8) (10 11) (11 12) (12 1))
20	((1 2) (2 3) (4 5) (5 8) (6 5) (7 1) (8 9) (9 10) (10 11) (11 12) (12 1))	51	((1 2) (2 3) (3 4) (4 5) (5 8) (6 7) (7 1) (9 8) (10 9) (11 12) (12 1))
21	((1 2) (2 3) (4 5) (5 6) (6 7) (7 1) (8 9) (9 10) (10 11) (11 12) (12 1))	**52**	((1 2) (2 3) (3 4) (4 5) (5 8) (6 5) (7 1) (9 8) (10 9) (11 12) (12 1))
22	((1 2) (2 3) (4 5) (5 6) (6 7) (7 1) (8 5) (9 10) (10 11) (11 12) (12 1))	**53**	((1 2) (2 3) (3 4) (4 5) (5 8) (6 7) (7 1) (8 9) (10 11) (11 12) (12 1))
23	((1 2) (2 3) (4 5) (5 6) (6 7) (7 1) (8 5) (9 8) (10 11) (11 12) (12 1))	54	((1 2) (2 3) (3 4) (4 5) (5 8) (6 5) (7 1) (8 9) (10 11) (11 12) (12 1))
**24**	((1 2) (2 3) (4 5) (5 6) (6 7) (7 1) (8 5) (9 8) (10 9) (11 12) (12 1))	55	((1 2) (2 3) (3 4) (4 5) (5 8) (6 7) (7 1) (8 9) (10 9) (11 12) (12 1))
**25**	((1 2) (2 3) (3 4) (5 8) (6 7) (7 1) (8 9) (9 10) (10 11) (11 12) (12 1))	**56**	((1 2) (2 3) (3 4) (4 5) (5 8) (6 5) (7 1) (8 9) (10 9) (11 12) (12 1))
26	((1 2) (2 3) (3 4) (5 8) (6 5) (7 1) (8 9) (9 10) (10 11) (11 12) (12 1))	**57**	((1 2) (2 3) (3 4) (4 5) (5 8) (6 7) (7 1) (8 9) (9 10) (11 12) (12 1))
27	((1 2) (2 3) (3 4) (5 6) (6 7) (7 1) (8 9) (9 10) (10 11) (11 12) (12 1))	**58**	((1 2) (2 3) (3 4) (4 5) (5 8) (6 5) (7 1) (8 9) (9 10) (11 12) (12 1))
28	((1 2) (2 3) (3 4) (5 6) (6 7) (7 1) (8 5) (9 10) (10 11) (11 12) (12 1)	**59**	((1 2) (2 3) (3 4) (4 5) (5 8) (6 7) (7 1) (8 9) (9 10) (10 11) (12 1))
29	((1 2) (2 3) (3 4) (5 6) (6 7) (7 1) (8 5) (9 8) (10 11) (11 12) (12 1))	**60**	((1 2) (2 3) (3 4) (4 5) (5 8) (6 5) (7 1) (8 9) (9 10) (10 11) (12 1))
**30**	((1 2) (2 3) (3 4) (5 6) (6 7) (7 1) (8 5) (9 8) (10 9) (11 12) (12 1))	**61**	((1 2) (2 3) (3 4) (4 5) (5 8) (6 7) (7 1) (8 9) (9 10) (10 11) (11 12))
**31**	((1 2) (2 3) (3 4) (4 5) (6 7) (7 1) (8 9) (9 10) (10 11) (11 12) (12 1))	**62**	((1 2) (2 3) (3 4) (4 5) (5 8) (6 5) (7 1) (8 9) (9 10) (10 11) (11 12))

[a] Sequential numbering groups the node-ponds corresponding to any one surface species on which the different **Σ-T**s end: first and last label for each node-pond is bold. As an example, **Σ-T** graphs from **1** to **12** are concerning node 1, that is, species σO; from **13** to **18** node 2, that is, species I2; and so on up to **61** and **62** regarding node 12, that is, species I12. [b] Each **Σ-T** graph is represented by one tuple, ((n m)···), being (n m) the edge/elementary-event between nodes n/surface-reagent and m/surface-product.

Optimized geometries and energetics (SCF energy plus zero-point vibrational contribution, E_{zpv}, calculated with DFT-M06L, Stuttgart RSC ECP on Mn, cc-pVDZ on H,C,O) of the species involved in the investigated processes

CO E_{zpv} = -113.303978 a.u.

H_2 E_{zpv} = -1.156956 a.u.

CO_2 E_{zpv} = -188.582092 a.u.

H_2O E_{zpv} = -76.392563 a.u.

O_2 E_{zpv} = -150.319420 a.u.

Mn_4O_8 (1) E_{zpv} = -1019.565304 a.u.

O	-2.286113	-2.008273	-0.748816
Mn	-0.632091	-2.451256	-0.338981
O	0.047347	-3.641162	-1.086013
Mn	-2.088995	-0.181889	-0.787368
O	-2.004715	0.201512	1.186261
Mn	-0.061563	-0.314924	1.269275
O	-0.146923	-0.699135	-0.704487
O	-1.929690	1.647054	-0.862622
Mn	-1.520161	1.953794	0.822116
O	-2.201176	3.143177	1.568486
O	0.134274	1.511421	1.232383
O	-0.220925	-2.143655	1.345286

$Mn_4O_8(CO)$ (2) E_{zpv} = -1132.878866 a.u.

O	1.678508	-1.409690	0.834173
Mn	2.286685	0.029110	0.010802
O	3.746843	0.035249	-0.543025
Mn	0.002235	-1.453636	0.093500
O	-0.880540	0.028440	1.057363
Mn	-0.069126	1.432443	-0.008144
O	0.851221	-0.066423	-1.120556
O	-1.668883	-1.595346	-0.676466
Mn	-2.360004	-0.061948	-0.162979
O	-3.585669	-0.049115	0.860358
O	-1.734603	1.452967	-0.802544
O	1.621193	1.512456	0.698589
C	-3.748151	-0.163387	-1.838667
O	-4.475873	-0.215824	-2.703583

Transition state (2)→(3) E_{zpv} = -1132.859007 a.u.

O	0.066276	-3.654548	-0.847447
Mn	-0.878547	-2.547330	0.005139
O	-0.440508	-2.061292	1.597200
Mn	-0.139011	-0.228656	1.357101
O	0.094284	1.579361	1.302478
Mn	-1.541297	2.042177	0.797296
O	-1.783398	1.692520	-0.924190
Mn	-1.966709	-0.118616	-0.811564
O	-0.156266	-0.661836	-0.508105
O	-2.346330	-1.946482	-0.663719
O	-2.116613	0.370000	1.197808
O	-2.217812	3.296056	1.431350
C	-1.125319	-4.449459	0.116694
O	-1.263511	-5.594964	0.175058

$Mn_4O_7(CO_2)$ (3) E_{zpv} = -1132.946760 a.u.

O	0.000000	0.000000	0.000000
Mn	0.000000	0.000000	1.739741
O	1.364832	0.000000	2.488018
Mn	-1.334664	1.235221	-0.365857
O	-2.977157	0.393167	0.183902
Mn	-2.578286	0.552708	2.313339
O	-1.006184	1.535162	1.791412
O	-2.407934	2.648677	-0.764633
Mn	-3.732899	2.057709	0.369072
O	-4.990086	3.821736	0.613945
O	-3.959829	1.738831	2.169117
O	-1.361029	-0.843322	2.446209
C	-5.267480	4.163197	1.711125
O	-5.568176	4.547726	2.755877

Mn_4O_7 (4) E_{zpv} = -944.341176 a.u.

O	-2.286113	-2.008273	-0.748816
Mn	-2.088995	-0.181889	-0.787368
Mn	-1.520161	1.953794	0.822116
O	0.134274	1.511421	1.232383
Mn	-0.061563	-0.314924	1.269275
O	-0.220925	-2.143655	1.345286
Mn	-0.632091	-2.451256	-0.338981
O	-0.146923	-0.699135	-0.704487
O	0.047347	-3.641162	-1.086013
O	-2.004715	0.201512	1.186261
O	-1.929690	1.647054	-0.862622

Mn_4O_9 (5) E_{zpv} = -1094.696034 a.u.

O	-2.426200	-2.050633	-1.080027
Mn	-1.998349	-0.351681	-1.135336
O	-1.912376	1.612313	-1.087954
Mn	-1.876180	1.778791	0.593795
O	-0.346402	1.486311	1.250667
Mn	-0.439096	-0.478009	1.202683
O	-0.736326	-2.188008	1.448511
Mn	-0.939698	-2.864178	-0.284513
O	-0.230580	-0.722615	-0.643283
O	-2.461573	0.177824	0.900371
O	-2.745240	2.901942	1.234682
O	0.385288	-4.329782	-0.469128
O	-0.336424	-4.270210	-1.547598

$Mn_4O_9(CO)$ (6) E_{zpv} = -1208.015384 a.u.

O	-2.733721	-2.018016	-0.872831
Mn	-1.172537	-2.783178	-0.125805
O	-0.443118	-3.752547	-1.502200
O	0.327414	-3.815789	-0.349634
Mn	-2.193730	-0.362030	-1.023013
O	-2.448246	0.317976	0.895929
Mn	-0.614986	-0.491095	1.338510
O	-0.447536	-1.038048	-0.515090
O	-1.788200	1.479892	-1.216817
Mn	-1.659588	1.885012	0.454648
O	-2.312693	3.208495	0.963457
O	-0.188144	1.349105	1.177269
O	-1.043433	-2.156201	1.654969
C	-2.189990	-4.482597	0.461193
O	-2.746819	-5.415487	0.782186

Transition state (6)→(7) E_{zpv} = -1207.998139 a.u.

O	-2.812945	-1.895831	-0.736068
Mn	-2.157881	-0.151676	-1.056131
O	-1.783165	1.612406	-1.306392
Mn	-1.585419	2.078101	0.396045
O	-0.037743	1.473990	1.023754
Mn	-0.482932	-0.284666	1.178624
O	-1.073509	-2.034032	1.584529
Mn	-1.526720	-2.670476	0.070036
O	-0.441994	-0.800349	-0.631429
O	-2.378152	0.538117	0.898175
O	-2.132082	3.452787	0.887331
O	-0.619366	-3.937641	-0.685695
O	-1.625840	-5.146487	-0.002710
C	-2.458429	-4.387037	0.666460
O	-3.392866	-4.668054	1.350290

$Mn_4O_8(CO_2)$ (7) E_{zpv} = -1208.169266 a.u.

O	-2.314688	-2.029287	-0.825143
Mn	-2.082952	-0.173990	-0.889735
O	-1.831112	1.658388	-0.877986
Mn	-1.509595	1.949904	0.816016
O	0.124661	1.491456	1.290915
Mn	-0.158393	-0.321182	1.327263
O	-0.308768	-2.159005	1.322490
Mn	-0.671654	-2.442058	-0.373060
O	-0.229101	-0.680507	-0.740259
O	-2.012124	0.214334	1.219547
O	-2.212669	3.157307	1.516492
O	-4.220189	-0.124602	-1.369167
O	0.043706	-3.622359	-1.104998
C	-4.920672	-1.070597	-1.275723
O	-5.645432	-1.964162	-1.198830

$Mn_4O_9(H_2)$ (8) E_{zpv} = -1095.860908 a.u.

O	-2.470606	-2.035395	-1.026297
Mn	-0.995870	-2.840729	-0.237410
O	-0.310794	-4.158311	-1.553472
O	0.412756	-4.216490	-0.476727
Mn	-2.031398	-0.330709	-1.089193
O	-2.428282	0.191878	0.886829
Mn	-0.474948	-0.448148	1.211862
O	-0.249865	-0.727488	-0.634667
O	-1.897365	1.576949	-1.132210
Mn	-1.828269	1.840569	0.564426
O	-2.642350	3.024638	1.175999
O	-0.294245	1.456035	1.236410
O	-0.775564	-2.163429	1.475998
H	-2.681486	-4.387112	0.699651
H	-2.201929	-4.944478	0.508132

Transition state (8)→(9) E_{zpv} = -1095.820289 a.u.

O	1.358547	-1.493272	0.876418
Mn	2.209114	-0.031626	0.216481
O	3.517960	-0.673532	-0.891300
O	3.783722	0.714578	-0.711588
Mn	-0.194636	-1.416251	0.012343
O	-1.161013	-0.000965	1.118662
Mn	-0.148031	1.417528	-0.006940
O	0.640903	0.001571	-1.028758
O	-1.888657	-1.412775	-0.803947
Mn	-2.513489	0.028180	-0.076391
O	-3.975074	0.016877	0.468268
O	-1.884350	1.468388	-0.775521
O	1.351650	1.502010	0.879665
H	3.502279	0.122535	1.343572
H	3.924526	0.594038	0.546725

$Mn_4O_8(H)(OH)$ (9) E_{zpv} = -1095.851607 a.u.

O	1.319830	-1.522491	0.894812
Mn	2.236532	-0.073602	0.469483
O	3.588949	-0.732135	-0.590700
O	3.784419	0.714561	-0.696709
Mn	-0.204202	-1.398187	-0.072357
O	-1.204496	-0.029772	1.109008
Mn	-0.143957	1.375789	-0.045594
O	0.566503	0.002983	-1.074821
O	-1.913915	-1.402260	-0.856364
Mn	-2.528976	0.029003	-0.087404
O	-3.992046	0.010521	0.456192
O	-1.901964	1.473603	-0.779275
O	1.302953	1.448468	0.944658
H	3.086211	-0.058980	1.747532
H	4.570770	0.860895	-0.132653

Transition state (9)→(10) E_{zpv} = -1085.833848 a.u.

O	-0.018778	0.043518	0.034896
Mn	-0.011398	0.026280	1.840839
Mn	1.793589	-0.007763	3.887325
O	2.204965	1.661260	3.840863
Mn	1.978420	1.931700	1.962743
O	2.128569	2.034735	0.214240
Mn	0.444579	1.682276	-0.426750
O	0.127601	1.899564	1.867130
O	-1.185101	2.397373	-1.052753
O	-0.209428	3.483986	-0.991149
O	2.026984	-0.158405	2.122299
O	0.091780	-0.286710	3.699122
O	2.607884	-0.970532	4.807644
H	0.945589	1.270117	-1.809077
H	-0.526599	4.357996	-1.265315

$Mn_4O_8(H)(OH)$ (10) E_{zpv} = -1095.852218 a.u.

O	1.337527	-1.505909	0.913462
Mn	2.255428	-0.062073	0.494230
O	3.524184	-0.714440	-0.659715
O	3.600708	0.719715	-0.922264
Mn	-0.169484	-1.372961	-0.086145
O	-1.186428	-0.046775	1.098579
Mn	-0.116922	1.393904	0.002100
O	0.617232	0.061220	-1.070533
O	-1.852641	-1.371562	-0.909405
Mn	-2.497297	0.040994	-0.120451
O	-3.973601	0.002734	0.383481
O	-1.858237	1.507931	-0.757965
O	1.323597	1.448246	1.002993
H	3.155073	-0.064466	1.732664
H	3.101845	0.797884	-1.763264

Transition state (10)→(11) E_{zpv} = -1095.814053 a.u.

O	1.371333	-1.579676	0.839442
Mn	2.184394	-0.123550	0.238057
O	3.681909	0.590337	-1.165885
O	3.561715	-0.827634	-0.852987
Mn	-0.247238	-1.450769	0.000390
O	-1.852817	-1.396546	-0.912627
Mn	-2.518227	0.040701	-0.147280
O	-4.023849	0.047990	0.271220
Mn	-0.161336	1.390393	0.210926
O	-1.160916	-0.106678	1.118374
O	0.713036	0.048608	-1.014546
O	-1.786779	1.542792	-0.679010
O	1.427446	1.289908	1.068819
H	3.442738	-0.397816	1.268770
H	3.239647	0.633674	-2.039392

$Mn_4O_7(OH)_2$ (11) E_{zpv} = -1095.931944 a.u.

O	1.320065	-1.508597	0.683593
Mn	-0.207281	-1.355204	-0.171793
Mn	-2.558680	0.042906	-0.037957
O	-1.955562	1.516799	-0.680312
Mn	-0.172848	1.380618	0.020502
O	1.347296	1.378062	0.884127
Mn	2.302470	-0.041469	0.138625
O	3.778051	-1.084738	-0.604993
O	3.614552	1.097504	-0.747824
O	0.471932	0.090206	-1.187895
O	-1.165968	-0.046798	1.097518
O	-1.989634	-1.353005	-0.867171
O	-3.997211	0.020533	0.571878
H	3.522889	1.046130	-1.715920
H	4.605427	-1.023115	-0.097280

Transition state (11)→(12) E_{zpv} = -1095.904937 a.u.

O	1.230900	-1.503908	0.885242
Mn	2.225928	-0.077699	0.327750
O	0.353182	0.016808	-1.044347
Mn	-0.318363	1.237964	0.089697
O	-1.262821	0.072690	1.123488
Mn	-2.706886	-0.002820	-0.085667
O	-4.196434	0.020016	0.390472
Mn	-0.312868	-1.471422	-0.014882
O	-2.065642	-1.503573	-0.704197
O	-2.019401	1.443499	-0.760267
O	1.200285	1.423121	0.895237
O	3.619586	-1.098996	-0.493145
O	3.887595	1.020529	-0.331843
H	3.799241	1.381040	-1.230604
H	4.294862	-0.092052	-0.415962

$Mn_4O_8(H_2O)$ (12) E_{zpv} = -1095.985411 a.u.

O	1.626464	-1.127321	0.901153
Mn	2.133933	0.168682	-0.165090
O	3.273186	-0.096497	-1.244648
O	3.632842	0.831772	1.217302
Mn	-0.100632	-1.340621	0.311417
O	-0.966314	0.363764	1.095940
Mn	-0.291236	1.419058	-0.509536
O	0.558734	-0.236283	-1.161909
O	-1.846881	-1.626633	-0.189162
Mn	-2.499653	-0.028956	0.172852
O	-3.898126	0.083936	0.860429
O	-2.032195	1.168006	-1.038175
O	1.429906	1.783967	0.028020
H	3.383425	1.751699	1.403494
H	4.452439	0.894382	0.701248

References

(1) Eyring, H.; Walter, J.; Kimball, G. E. *Quantum Chemistry*, 2nd ed.; John Wiley & Sons, Inc.: New York, NY, 1946; pp 299–331.

(2) Grimme, S. Supramolecular Binding Thermodynamics by Dispersion-Corrected Density Functional Theory. *Chem. Eur. J.* **2012**, *18*, 9955–9964.

(3) Luchini, G.; Alegre-Requena, J. V.; Funes-Ardoiz, I.; Paton, R. S. Automated Thermochemistry for Heterogeneous Computational Chemistry Data. *F1000Research* **2020**, *9*, 291.

(4) Arena, F.; Ferrante, F.; Cajumi, A.; Cannilla, C.; Todaro, S.; Bertini, M.; Gueci, L.; Bonura, G.; Pàszti, Z.; Duca, D. Molecular Dynamics and Kinetic Modelling of the CO and H_2 Oxidation Pattern of a Composite $MnCeO_x$ Catalyst. *Chem. Eng. J.* **2025**, *505*, 158677.

(5) (a) Arena, F.; Ferrante, F.; Di Chio, R.; Bonura, G.; Frusteri, F.; Frusteri, L.; Prestianni, A.; Morandi, S.; Martra, G.; Duca, D. DFT and Kinetic Evidences of the Preferential CO Oxidation Pattern of Manganese Dioxide Catalysts in Hydrogen Stream (PROX). *Appl. Catal. B: Environ.* **2022**, *300*, 120715; (b) Gueci, L.; Arena, F.; Todaro, S.; Bonura, G.; Cajumi, A.; Bertini, M.; Ferrante, F.; Nania, C.; Duca, D. CO-PROX on MnO_2 Catalysts: DFT-Based Microkinetic and Experimental Macrokinetic Approaches. *Catal. Today* **2024**, *434*, 114698.
